# Exploring the shared genetic architecture between periodontitis and cardiovascular disease

**DOI:** 10.1038/s41405-026-00421-4

**Published:** 2026-03-31

**Authors:** Ting Jin, Jinfeng Lin, Peng Zhang, Yu Lu, Shenguo Chen, Haisheng Lin

**Affiliations:** 1 Taizhou Hospital of Zhejiang Province, affiliated with Wenzhou Medical University, Taizhou, Zhejiang China; 2 Enze Hospital, Taizhou Enze Medical Center (Group), Taizhou, Zhejiang China

**Keywords:** Periodontitis, Oral pathology

## Abstract

**Background:**

Epidemiological evidence supports the association between periodontitis and cardiovascular diseases (CVD); however, their shared genetic mechanisms remain inadequately defined. This study elucidates their genetic architecture by identifying shared risk loci and associated genes.

**Methods:**

This study employs Mendelian randomization (MR) to investigate bidirectional causal relationships between periodontitis and five types of CVD based on genome-wide association study (GWAS) summary data. Cross-trait analyses were applied to examine genetic correlations across trait pairs, identifying pleiotropic loci and associated genes. Functional annotation and tissue-specificity analyses elucidate their biological functions.

**Results:**

Bidirectional and multivariable MR analyses confirmed that the association between CVD and periodontitis is not driven by a direct causal relationship. Additionally, the genetic correlation between these disorders underscores the importance of investigating their shared genomic architecture. Colocalization analysis identified significant shared causal variants at loci 4p14 and 15q25.1. At the gene level, seven unique pleiotropic genes (e.g., *CD151*, *POLR2L*, and *HLA-DQA1*) were annotated. Pathway analysis revealed that these genetic architectures likely mediate cross-disease interactions through an inflammation-metabolism regulatory axis (Inflammatory Response and Cholesterol Metabolism Pathway). Tissue enrichment analyses demonstrated that pleiotropic signals, from SNP to gene levels, were significantly enriched in immune-related tissues and disease-relevant sites like the heart.

**Conclusion:**

This study reveals a shared genetic basis between periodontitis and five types of CVD, suggesting potential underlying mechanisms. However, based on summary-level data, it remains unclear whether this association represents direct biological genetic determinants or indirect pathways mediated by shared environmental or behavioral risk factors. Future studies utilizing individual-level data with covariate adjustments are needed to further investigate these relationships.

## Introduction

Noncommunicable diseases (NCD) contribute to 41 million annual deaths globally, constituting 74% of all mortality as epidemiological surveillance [1]. These diseases impose a significant global health burden and significantly hinder sustainable development goals through productivity losses, establishing their containment as a 21st-century imperative for public health systems worldwide. Cardiovascular diseases (CVD), including hypertension (HTN), myocardial infarction (MI), atherosclerosis (AS), and coronary heart disease (CHD), constitute the predominant cause of NCD-related mortality worldwide, accounting for 17.9 million annual deaths (45% of total NCD-related fatalities) [2]. Although CVD mortality rates have declined in recent decades, aging populations are projected to intensify the burden in the future [3]. Periodontitis, another widespread NCD, affects nearly one billion individuals globally and ranks as the sixth most prevalent disease worldwide [4]. The American Academy of Periodontology (AAP) recognized periodontitis as a risk modifier for CVD via oral microbial translocation. This biological mechanism triggers systemic inflammation (elevated C-reactive protein) and oxidative stress, accelerating atherogenesis through endothelial dysfunction and lipid deposition [5]. In support of this hypothesis, pathogen-derived Deoxyribonucleic acid and immunologic components, including viable *Porphyromonas gingivalis* and *Aggregatibacter actinomycetemcomitans*, were detected in AS-associated thrombotic specimens, providing histopathology evidence for the established periodontitis-cardiovascular axis [6, 7].

Mendelian randomization (MR) uses genetic variation, randomly assigned before birth, as a natural experiment to infer potential causal relationships, which minimizes confounding factors when assessing the causal link between exposures affected by these genetic variations and the outcome of interest [8, 9]. Prior investigations employing MR to elucidate causal associations between periodontitis and CVD have yielded conflicting evidence. For example, Zhang et al. identified a positive association between periodontitis and small vessel stroke [odds ratio (OR) = 1.15; 95% confidence interval (CI) = 1.00–1.33; *P* = 0.049]; in contrast, Steven et al. reported no significant association between periodontitis and any stroke [10, 11]. Genome-wide association studies (GWAS) have revealed pleiotropic connections between periodontitis and CVD. For example, the pleiotropic locus 9p21.3 (*CDKN2B-AS1*), which is associated with coronary artery disease, type 2 diabetes, ischemic stroke, and Alzheimer’s disease, is also correlated with elevated C-reactive protein levels in periodontitis, highlighting shared pathophysiological pathways [12]. Additionally, Schaefer et al. identified plasminogen as a common genetic risk factor for CHD and periodontitis [13]. Although a significant association exists between periodontitis and CVD, research on the pleiotropic genetic mechanisms driving their co-occurrence remains inadequate, necessitating urgent dissection of their shared genetic architecture.

This study aims to systematically explore the shared genetic architecture between periodontitis and five types of CVD. Notably, the use of summary-level GWAS data precludes adjustment for confounding factors such as environmental and behavioral influences. Therefore, the observed associations may arise either from direct biological mechanisms or indirect pathways mediated by shared environmental exposures.

## Materials and methods

### Study design

**Fig. 1:**
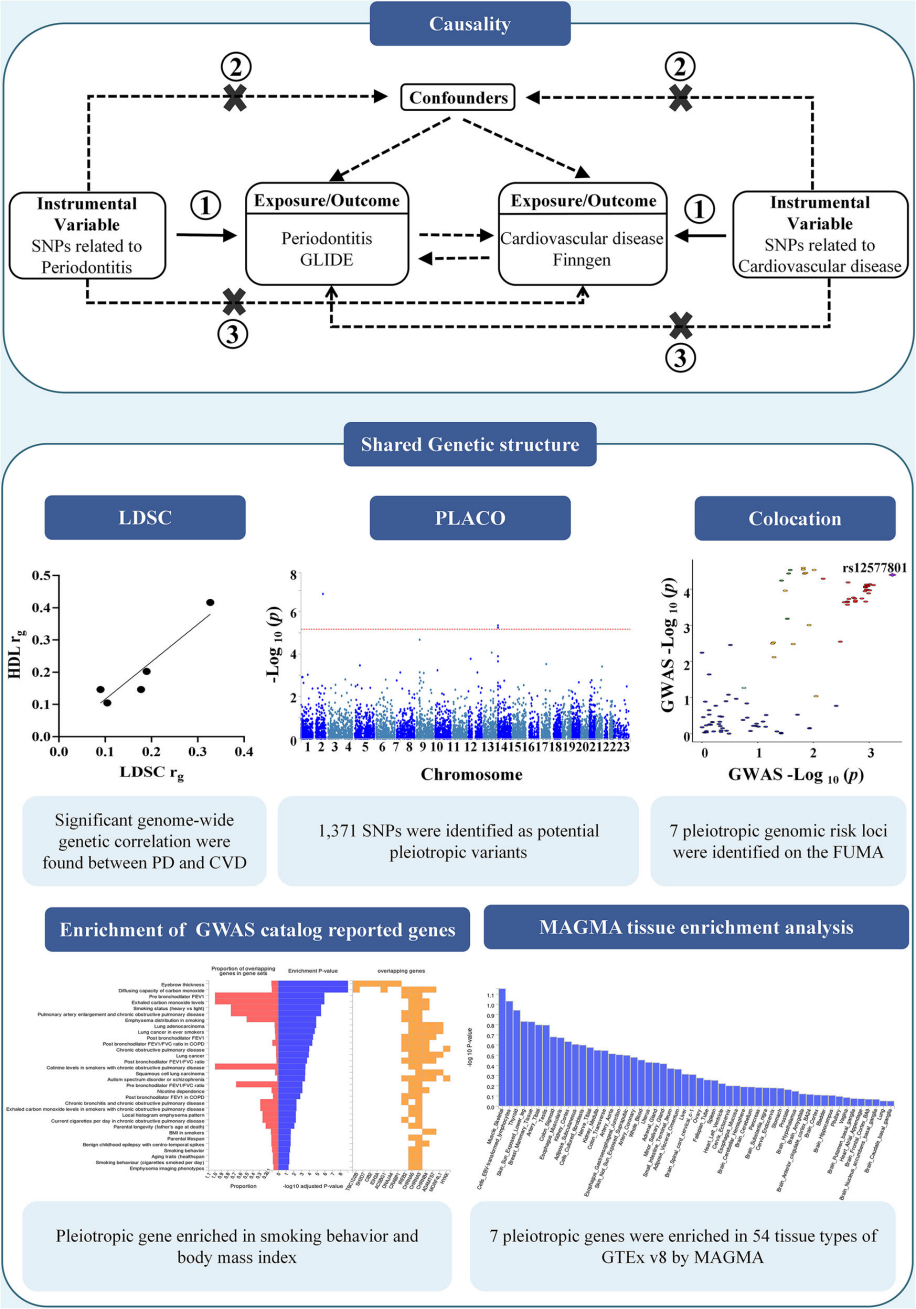
Study workflow. Note: 1, “relevance” assumption, the IVs are robustly associated with the exposure; 2, “exclusion restriction” assumption, the IVs are not related to confounding factors; 3, “exclusion restriction” assumption, the IVs do not affect the outcome via a pathway other than exposure. LDSC Linkage Disequilibrium Score Regression, GWAS genome-wide association study, PLACO pleiotropic analysis under the composite null hypothesis, MAGMA multi-marker analysis of GenoMic annotation.

As illustrated in Fig. 1, this study aims to apply MR analysis to assess the bidirectional causal associations between periodontitis and cardiovascular diseases (CVD, HTN, MI, AS, and CHD). Subsequently, various statistical genetic methods were employed to explore the shared genetic mechanisms between trait pairs. Specifically, within the pleiotropy analysis framework, Linkage Disequilibrium Score Regression (LDSC) and High-Definition Likelihood (HDL) were applied to assess genetic correlations between trait pairs. Subsequently, Pleiotropy Analysis under the Composite null hypothesis (PLACO) identified shared pleiotropic loci at the single-nucleotide polymorphism (SNP) level, followed by functional mapping and annotation of genetic associations using the functional mapping and annotation of genetic associations (FUMA) platform, followed by phenotype-associated and tissue-specific enrichment analyses. This study followed the STROBE-MR guidelines [14]. All participants in the original studies provided informed consent and received an ethical review.

### GWAS data sources

We obtained GWAS summary statistics for European ancestry populations from public repositories. Details of the data sources were described in Supplementary Table 1. The GWAS summary statistics for periodontitis (single_trait_ALL_EUR) were derived from the Gene‒Lifestyle Interactions in Dental Endpoints (GLIDE) consortium, which included 17,353 cases and 28,210 controls with clinically defined periodontitis [15]. Periodontitis cases were defined using criteria from the Centers for Disease Control and Prevention (CDC) and the AAP [16]. Additionally, we accessed GWAS data for five types of CVD from the FinnGen database (R11), including CVD (Ncases = 221,781, Ncontrols = 231,952), HTN (Ncases = 137,312, Ncontrols = 316,345), AS (Ncases = 17,832, Ncontrols = 423,324), CHD (Ncases = 51,098, Ncontrols = 402,635), and MI (Ncases = 28,546, Ncontrols = 378,019) [17]. The FinnGen study, a significant genomics project, has analyzed over 500,000 Finnish biobank samples, correlating genetic variants with health data to elucidate disease mechanisms and predispositions [17]. Given the high comorbidity rate between periodontitis and CVD, publicly available summary-level data do not allow for the exclusion of individuals affected by both conditions. Consequently, phenotypic overlap may inflate estimates of genetic correlation and the strength of pleiotropic signals. This represents a recognized limitation of cross-trait analyses based on such data resources.

To evaluate the genetic information quality of the datasets used, we conducted a replication rate analysis of known loci. Specifically, for periodontitis, ten previously confirmed associated genes (e.g., *SIGLEC5*, *DEFA1*, *FCERG1*) were selected from published literature [18]. For various CVD subtypes, 100 validated SNPs were extracted from the Cardiovascular Disease Atlas (https://ngdc.cncb.ac.cn/). Subsequently, the *P*-values of these loci were retrieved from the corresponding summary statistics of this study, and their replication was assessed at distinct thresholds: genome-wide significance (*P* < 5 × 10⁻⁸).

### Mendelian randomization analysis

To satisfy the three core assumptions of MR analysis: correlation, exclusion restriction, and independence assumption, this study employs a series of stringent procedures to screen instrumental variables (IVs). Initially, SNPs strongly associated with the exposure at the genome-wide significance threshold (*P* < 5 × 10⁻⁸) were selected as IVs. If insufficient IVs were identified, a secondary threshold (*P* < 5 × 10⁻⁶) was applied to satisfy the relevance assumption. Subsequently, SNPs were pruned to minimize linkage disequilibrium (LD) within 1 Mb cluster windows (r² < 0.001), ensuring their independence [19]. Finally, the strength of IVs was validated by quantifying the phenotypic variation explained (R²) and evaluating the instrument strength using the F-statistic (F > 10) to mitigate weak instrument bias [20, 21]. Finally, to satisfy the exclusion assumption, SNP filtering was performed via the LDlink platform (https://ldlink.nih.gov), excluding variants strongly associated (*P* < 5 × 10⁻⁶) with known confounders using LD analysis and removing palindromic SNPs from the candidate set (Supplementary Fig. 1).

Before conducting the MR analysis, the Mendelian Randomization with Latent Sample Overlap and Pleiotropy (MRlap) method was employed to detect potential sample overlap and evaluate its possible bias in the results. This package utilizes cross-trait LDSC to approximate the degree of sample overlap, thereby enabling the assessment and correction of bias introduced by sample overlap in the MR analysis [22, 23]. An online platform (https://sb452.shinyapps.io/power/) was employed to compute the a priori statistical power [24].

In the primary analysis, the inverse-variance weighted (IVW) model was used as the main analytical approach, treating each SNP as a valid natural experiment to minimize bias and maximize statistical power. Sensitivity analyses were conducted using the MR-Egger regression [25], the weighted median, the simple mode, the weighted mode, and the Contamination Mixture (MRConMix) to assess the directional consistency of effect estimates derived from IVW [26]. These alternative approaches permit the presence of horizontal pleiotropy, albeit with reduced statistical power compared to the IVW method. MRConMix robustly executes MR analysis even with invalid IVs, demonstrating the lowest mean squared error. Cochran’s Q test assessed heterogeneity among SNPs in the IVW estimates (*P* < 0.05 indicates significant heterogeneity) [27]. If considerable heterogeneity was detected, the IVW random-effects model was applied; otherwise, the fixed-effects model was utilized. MR-PLiotropic RESidual Sum and Outlier (MR-PRESSO) was implemented to detect outliers, and analyses were repeated after removing outliers when statistical significance was observed. Horizontal pleiotropy was evaluated via the MR-Egger intercept test. The Robust Adjusted Profile Score (MR-RAPS) and the MR-Lasso were employed as sensitivity analyses to assess the robustness of the causal effect estimates in the presence of horizontal pleiotropy [28, 29]. Furthermore, Steiger directionality testing was conducted to verify directionally concordant causal relationships between trait pairs, thereby mitigating bias from reverse causality. To correct for multiple comparisons, this study applied Storey’s q-value false discovery rate (FDR) correction in the final *P* estimates [30]. Additionally, the Causal Analysis Using Summary Effect Estimates (CAUSE) method was applied to improve the robustness of causal inference. By incorporating complete genome-wide summary data rather than solely genome-wide significant loci, CAUSE can better differentiate actual causal effects from correlated pleiotropy and account for uncorrelated horizontal pleiotropy, thus reducing false positives commonly encountered with other MR approaches. For this analysis, SNPs were included at an arbitrary value of *P* < 1 × 10^−5^, and statistical significance was set at *P* < 0.05 [31, 32]. Finally, building upon the traditional MR framework, a multivariable MR (MVMR) approach was implemented. This method incorporated multiple exposures simultaneously into the model to account for potential confounding, thereby improving the accuracy of the causal effect estimates [33].

### Genome-wide genetic correlation analysis

LDSC assessed genome-wide genetic correlations between periodontitis and five types of CVD. Precomputed LD scores from the 1000 Genomes Project Phase 3 European ancestry data were utilized for LDSC calculations [19]. The standard error (SE) was estimated via jackknife resampling within the LDSC framework to correct attenuation bias. Furthermore, no constraint was imposed on the LDSC intercept, enabling both residual confounding adjustment and detection of potential sample overlap between the two GWAS studies [34]. Compared to LDSC, the HDL method more effectively leverages GWAS summary statistics to estimate genetic correlations. Using 1,029,876 accurately imputed HapMap3 SNPs as a reference panel, HDL computes pairwise trait correlations, thereby validating LDSC results with enhanced resolution [35].

### Pleiotropic loci and gene analysis

PLACO was a statistical approach specifically designed to detect genetic pleiotropy, enabling the identification of shared genetic variants across multiple phenotypes. However, it cannot distinguish whether the observed associations arise from direct biological pleiotropy, indirect genetic effects mediated by correlated factors (e.g., smoking, obesity), or horizontal pleiotropy. This limitation is inherent to pleiotropy analyses based on summary-level GWAS data without adjustment for covariates. Initially, PLACO was applied to identify pleiotropic SNPs between trait pairs (*P*._*PLACO*_ < 5 × 10^–8^) [36]. These pleiotropic SNPs may play critical roles in disease pathogenesis, and placing them was essential for elucidating the shared genetic basis underlying the relationship between periodontitis and five types of CVD. The genomic control factor (λGC) was calculated to assess test statistic inflation in the PLACO analysis [37]. Subsequently, the FUMA was employed to perform functional annotation and gene mapping of these pleiotropic SNPs, with the major histocompatibility complex region (MHC) excluded from the analysis due to its pronounced linkage disequilibrium to minimize potential false positives [38]. Bayesian colocalization analysis was then performed on FUMA-annotated pleiotropic loci to identify primary shared risk loci for corresponding trait pairs within each pleiotropic region [39]. This analysis computes the posterior probabilities (PP) for five distinct hypotheses at each locus: (i) H0: Neither exposure nor outcome has a genetic association in this region; (ii) H1: Association with the exposure only; (iii) H2: Association with the outcome only; (iv) H3: Independent associations for both, driven by two distinct causal variants; (v) H4: Shared association for both, driven by one causal variant. Loci with PP.H4 > 0.7 were considered colocalization sites potentially harboring common causal variants, and the SNP with the highest PP.H4 was designated as the candidate causal variant. Fine-mapping was performed using eCAVIAR on large-scale GWAS summary statistics without individual genotype data to identify high-confidence genes and regulatory mechanisms [expression/splicing quantitative trait loci (eQTL/sQTL)] underlying the risk loci. For this analysis, a colocalization posterior probability (CLPP) greater than 0.1 was set as the significance threshold for GWAS-to-QTL-to-tissue combinations [40].

To further explore the shared mechanisms of pleiotropic loci, we performed gene-level genome-wide annotation of risk loci using Multi-marker Analysis of Genomic Annotation (MAGMA) [41]. This approach identifies pleiotropic genes by accounting for LD between markers and detecting multi-marker effects. Additionally, MAGMA gene-set analysis was conducted with 10,678 gene sets from the Molecular Signatures Database (MSigDB) to investigate the biological functions of lead SNPs [42]. To mitigate false-positive findings, Bonferroni correction was employed to adjust *P* for multiple testing (*P* < 0.05/10,678 = 4.68 × 10^–6^). We performed tissue-specific enrichment analyses of these pleiotropic genes across 54 Genotype-Tissue Expression (GTEx) tissues. The mean expression (log2-transformed) of all identified pleiotropic genes was calculated for each tissue, with tissue specificity tested through differentially expressed gene (DEG) analysis (predefined up/down-regulated DEGs based on the sign of t-statistics) [43]. Furthermore, stratified-LDSC (S-LDSC) regression was employed to validate the enrichment of SNP heritability for periodontitis and five types of CVD in specific tissues.

We used paired trait and immune cell GWAS data for multi-trait colocalization analysis (HyPrColoc) [44]. This advanced approach helps identify shared genetic variants between these conditions and immune cells, offering fresh insights into the immune system’s regulatory mechanisms in periodontitis and five types of CVD. Details on the summary GWAS dataset of immune cells are described in Supplementary Table 2.

### Summary‑based Mendelian randomization analysis

Based on the Summary-based Mendelian Randomization (SMR) method, GWAS data were integrated with eQTL studies to identify genes whose expression levels were associated with complex traits due to pleiotropy. The heterogeneity-in-dependent instrument (HEIDI) test was applied to distinguish whether the association between gene expression and the phenotype originated from pleiotropy or linkage disequilibrium by assessing heterogeneity [45]. A non-significant HEIDI result (*P* > 0.05) was interpreted as evidence that the association was likely mediated by a shared causal genetic variant. Conversely, a significant HEIDI result (*P* < 0.05) indicated heterogeneity, suggesting that the genetic association might be due to linkage disequilibrium [46]. To investigate the relationship between the genes and the phenotype, eQTL data from two sources were utilized: whole blood data from GTEx V8 (comprising 17,382 samples) and cis-eQTL data from the eQTLGen consortium (comprising 31,684 blood samples) [47, 48]. This approach elucidates gene regulatory mechanisms mediating genotype-phenotype relationships, ultimately facilitating drug target discovery.

### Software

We calculated the main statistics via R (v3.5.3) and analyzed the LDSC and S-LDSC with “LDSC” software (v1.0.1). PLACO was run with the “PLACO” package, and Bayesian colocalization was performed via “coloc” (v5.2.1) and “HyPrColoc” (v1.0). The functional analysis relied on FUMA (https://fuma.ctglab.nl/snp2gene), while MAGMA software was used for gene and gene set analyses. Two-sample MR analyses utilized the “TwoSampleMR” (v0.5.6) package. The main code used in this research was available at: https://zenodo.org/records/17505061.

## Results

### Mendelian randomization analysis between periodontitis and CVD

**Fig. 2:**
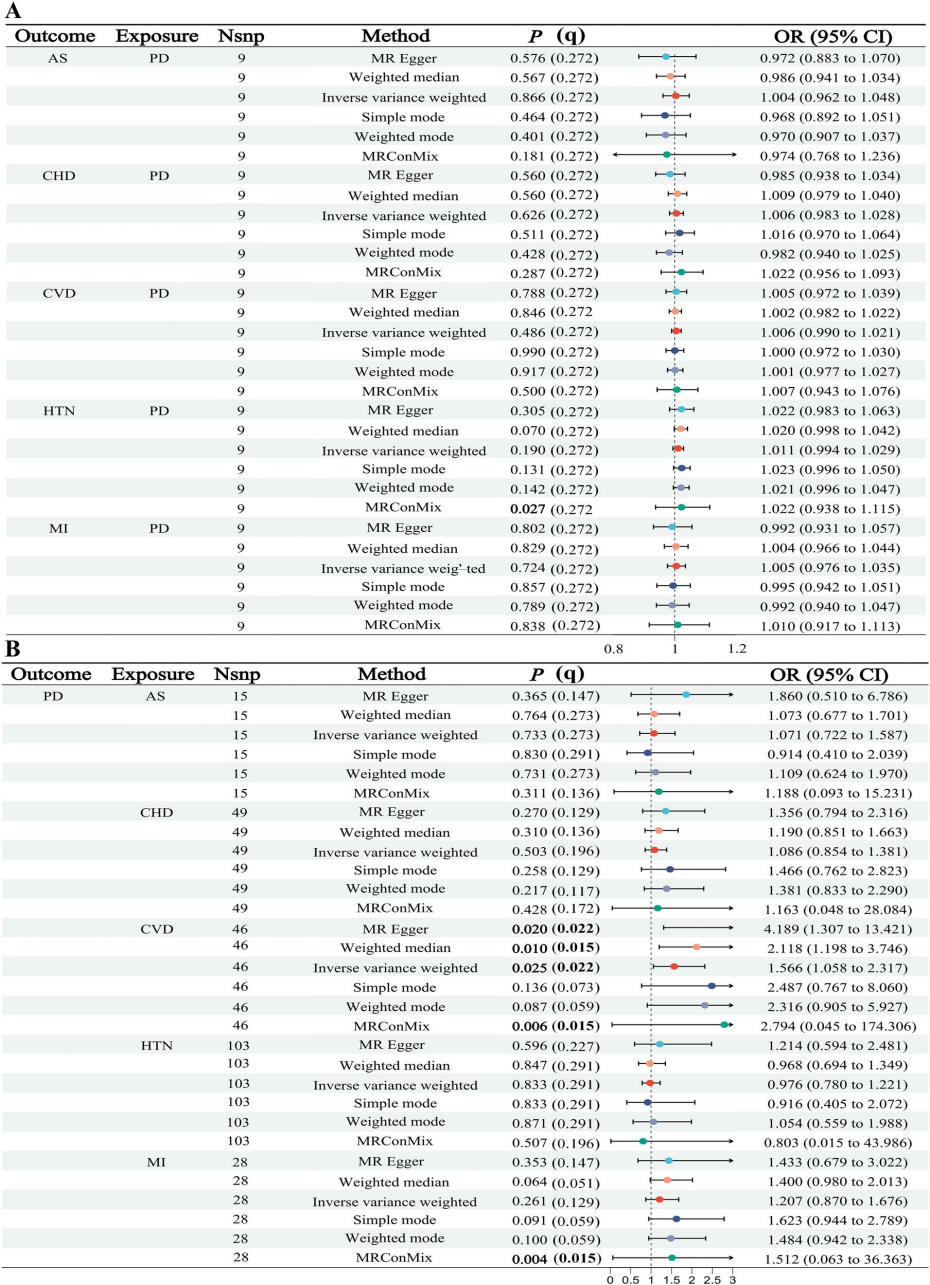
Bidirectional MR analyses for the causal associations between PD and five types of CVD. **A** MR analyses for the causal association of PD with five types of CVD. **B** MR analyses for the causal association of five types of CVD with PD. Note: *P* *P*-value, q Storey’s *q*-value for false discovery rate control, OR Odds ratio, PD Periodontitis, CVD Cardiovascular diseases, HTN Hypertension, MI Myocardial infarction, AS Atherosclerosis disease, CHD Coronary heart disease.

The absence of significant bias from sample overlap between the periodontitis and five types of CVD GWAS datasets was confirmed by MRlap analysis (Supplementary Table 3). Supplementary Fig. 1 outlines the IVs selection process for periodontitis and five types of CVD. Post-screening, SNPs significantly associated with each phenotype were included as valid IVs: periodontitis (*n* = 9), AS (*n* = 15), CHD (*n* = 49), CVD (*n* = 46), HTN (*n* = 103), and MI (*n* = 28). The characteristics of these IVs were detailed in Supplementary Tables 4, 5, while Supplementary Table 6 describes SNPs linked to potential confounders. This study had adequate statistical power (OR = 1.2; *P* < 0.05) to investigate the causal relationships of CHD, CVD, HTN, and MI with periodontitis (Supplementary Table 7). In the MR analyses examining the effects of periodontitis on five types of CVD, no causal association was observed. However, results from MRConMix (OR = 1.022, 95% CI = 0.938–1.115, *P* = 0.027) and MR-Lasso (Beta = 0.019; *P* = 0.025) suggested a potential statistical association between periodontitis and HTN. The MRConMix (*P*_*(q)*_ = 0.272) result did not pass the Storey’s q test and showed a wide confidence interval (Fig. 2A & Supplementary Table 8). Neither the Cochran’s Q-test nor the MR-PRESSO analysis detected outliers (Supplementary Table 8), and visual inspections through scatter plots and funnel plots excluded potential influential variants (Supplementary Fig. 2). These findings suggest that, although a statistical association exists, with an ambiguous effect direction and a clinically modest effect size, a comprehensive evaluation alongside biological mechanisms is necessary.

In reverse MR analyses, the IVW method demonstrated a significant causal association between CVD and periodontitis (OR = 1.566, 95% CI = 1.058-2.317, *P*_*(q)*_ = 0.022), which was supported by the MR-Egger (OR = 4.189, 95% CI = 1.307–13.421, *P*_*(q)*_ = 0.022), the weighted median (OR = 2.118, 95% CI = 1.198–3.746, *P*_*(q)*_ = 0.015), the MR-RAPS (Beta = 0.459, *P* = 0.028), and the MR-Lasso (Beta = 0.448, *P* = 0.025) methods (Fig. 2B & Supplementary Table 8). In contrast, the CAUSE analysis (Beta = −0.180, *P* = 0.510) did not support a causal link between trait pairs (Supplementary Table 8). However, no significant causal associations were detected between periodontitis and four specific CVD: AS (OR = 1.071, 95% CI = 0.722–1.587, *P*_*(q)*_ = 0.273), CHD (OR = 1.086, 95% CI = 0.854–1.381, *P*_*(q)*_ = 0.196), HTN (OR = 0.976, 95% CI = 0.780–1.221, *P*_*(q)*_ = 0.291), or MI (OR = 1.207, 95% CI = 0.870–1.676, *P*_*(q)*_ = 0.129). While MR-RAPS (Beta = 0.296, *P* = 0.046) and MR-LASSO (Beta = 0.304, *P* = 0.015) suggested a statistically significant association between MI and periodontitis, this finding was influenced by heterogeneity (Cochran’s Q = 50.332, *P* = 0.004). After removing outliers using MR-PRESSO, a causal relationship emerged (Beta = 0.304, *P* = 0.015). This result aligns with the findings reported by Du et al. based on independent datasets, further supporting a potential causal relationship between MI and periodontitis [11]. The visualization results for each trait pair (leave-one-out test, scatter plot, forest plot, and funnel plot) were provided in Supplementary Fig. 3. MVMR analysis that accounted for smoking, body mass index (BMI), and type 2 diabetes showed no direct causal link between CVD and periodontitis (Supplementary Fig. 4). These findings suggest that their association was likely attributable to shared genetic and pathophysiological mechanisms.

### Genetic correlations between periodontitis and CVD

Using bivariate LDSC analysis, significant genetic correlations were observed between periodontitis and five types of CVD, with the strongest correlation (r_g_ = 0.327) identified for AS. Genetic covariance intercept analysis revealed minimal sample overlap between trait pairs, indicating limited genetic confounding effects. HDL analysis further validated these findings, confirming shared genetic correlation and enhancing confidence in the results (Table 1). This study examined the replication rate of known trait-associated SNPs (*P* < 5 × 10⁻⁸) to assess the robustness of genetic signals within the datasets used. The results showed that none of the ten previously reported loci reached genome-wide significance in the periodontitis dataset. In contrast, among the CVD datasets, the overall inclusion rate of known SNPs was 78%, with the proportion replicated at the genome-wide level ranging from 3% to 40% (Supplementary Table 9).

**Table 1:** Genetic correlation between periodontitis and cardiovascular diseases.

Trait pairs	LDSC			HDL	
	r_g_ (SE)	*P*	Int (SE)	r_g_ (SE)	*P*
PD-CVD	0.189 (0.042)	5 × 10^−6^	0.001 (0.006)	0.202 (0.048)	2 × 10^−5^
PD-HTN	0.104 (0.042)	0.013	0.009 (0.006)	0.105 (0.042)	0.014
PD-MI	0.177 (0.052)	7 × 10^−4^	0.001 (0.005)	0.146 (0.052)	0.004
PD-AS	0.327 (0.065)	5 × 10^−7^	0.003 (0.006)	0.416 (0.074)	2 × 10^−8^
PD-CHD	0.279 (0.098)	0.004	0.008 (0.005)	0.291 (0.094)	0.003

*LDSC* linkage disequilibrium score regression, *HDL* high-definition likelihood, *SE* standard error, *Int* Genetic Covariance Intercept, *P* *P* value, *PD* Periodontitis, *CVD* Cardiovascular disease, *HTN* Hypertension, *MI* Myocardial Infarction, *AS* Atherosclerosis, *CHD* Coronary heart disease.

### Identification of pleiotropic loci between periodontitis and CVD and tissue enrichment analysis

Given the significant genetic correlation between periodontitis and five types of CVD, PLACO was employed to detect pleiotropic loci across trait pairs (Supplementary Fig. 5). Across five trait pairs, this study identified 1371 potential pleiotropic SNPs (Supplementary Table 10). To ensure reliability, this study performed quality control assessments. The QQ plot showed no systematic deviation of observed from expected *P*, and λGC for all trait pairs were close to 1 (λGC = 0.931–0.981). Together, these diagnostics support the validity of the PLACO findings (Supplementary Fig. 6 & Table 11).

**Fig. 3:**
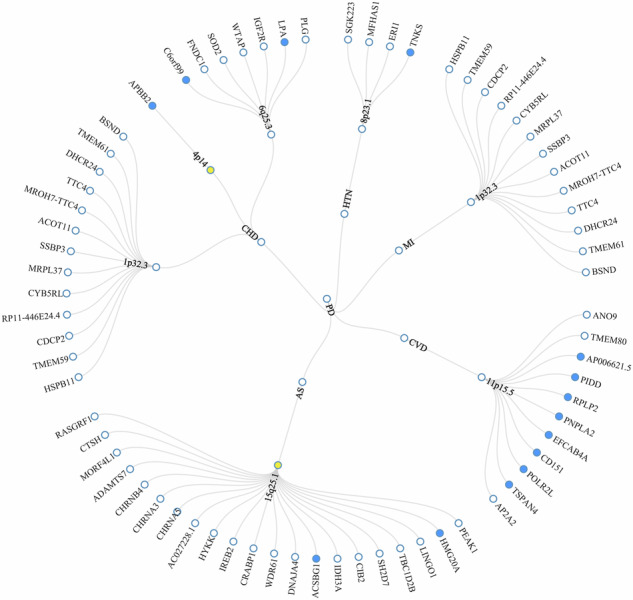
The circular diagram presents pleiotropic loci and genes identified by PLACO among the seven trait pairs. Shared loci identified by colocalization analysis are highlighted in orange; shared genes identified by MAGMA analysis are highlighted in blue. PD Periodontitis, CVD Cardiovascular disease, HTN Hypertension, MI Myocardial Infarction, AS Atherosclerosis, CHD Coronary heart disease.

Based on the above results, seven pleiotropic genomic risk loci were identified on the FUMA platform, spanning six unique chromosomal regions (Table 2 & Fig. 3). Supplementary Fig. 7 presents the results of the colocalization sensitivity analysis. Colocalization analysis further revealed two loci (15q25.1 and 4p14) with PP.H4 > 0.7, suggesting that periodontitis may share causal SNPs with AS and CHD within these genomic regions. For these two colocalized loci, eCAVIAR analysis identified genes significantly colocalized with AS (PSMA4, CHRNA5, RP11-650L12.2) and CHD (APBB2), all with CLPP > 0.01 (Supplementary Table 12). These genes showed significant enrichment in immune-related tissues (Spleen and Whole blood) and cardiac tissues (Artery_Coronary, Heart_Atrial_Appendage, and Heart_Left_Ventricle). Additionally, a PP.H3 > 0.7 (0.751) at 8p23.1 suggests that periodontitis and HTN are associated with SNPs in this region, but are driven by distinct causal variants (Table 2). Regional association plots for each trait pair are provided in Supplementary Figs. 8–12. Furthermore, leveraging the GWAS Catalog as a reference dataset, we detected significant associations between these genomic risk loci and phenotypes, such as smoking behavior and BMI (Supplementary Fig. 13). Annotation of seven lead SNPs using ANNOVAR revealed three variants in intergenic regions, two in downstream gene regions, with the remaining variants classified as intronic and 5’-untranslated region (5’-UTR) alterations (Table 2).

**Table 2:** Colocalized loci identified by colocalization analysis.

Trait pairs	Top SNP	Region	CHR:POS	Start-End	Functional annotation	*P*	CADD	PP.H3	PP.H4
PD-CVD	rs12577801	11p15.5	11:835877	828916-843097	UTR5	5.50E-07	1.363	0.018	0.256
PD-HTN	rs4841042	8p23.1	8:8807112	8020338-9486795	intergenic	4.30E-08	1.478	0.751	0.026
PD-MI	rs2746689	1p32.2	1:55354964	54391286-56339902	downstream	8.07E-09	4.478	0.626	0.009
PD-AS	rs12914385	15q25.1	15:78606381	77632340-79493215	intergenic	2.46E-12	2.976	0.109	0.852
PD-CHD	rs2746689	1p32.3	1:55354964	54391286-56339902	downstream	9.47E-07	4.478	0.127	0.732
PD-CHD	rs62412131	6q25.3	4:41021577	40987986-41143701	intronic	2.09E-07	1.689	0.626	0.009
PD-CHD	rs754748242	4p14	6:160307551	159325651-161118395	intergenic	1.01E-07	7.301	0.456	0.047

*PP.H3* posterior probability of H3, *PP.H4* posterior probability of H4; the locus boundary was defined as “chromosome: start-end”, *PD* Periodontitis, *CVD* Cardiovascular disease, *HTN* Hypertension, *MI* Myocardial Infarction, *AS* Atherosclerosis, *CHD* Coronary heart disease.

Lead SNP was the SNP with minimum *P* within the corresponding locus.

**Fig. 4:**
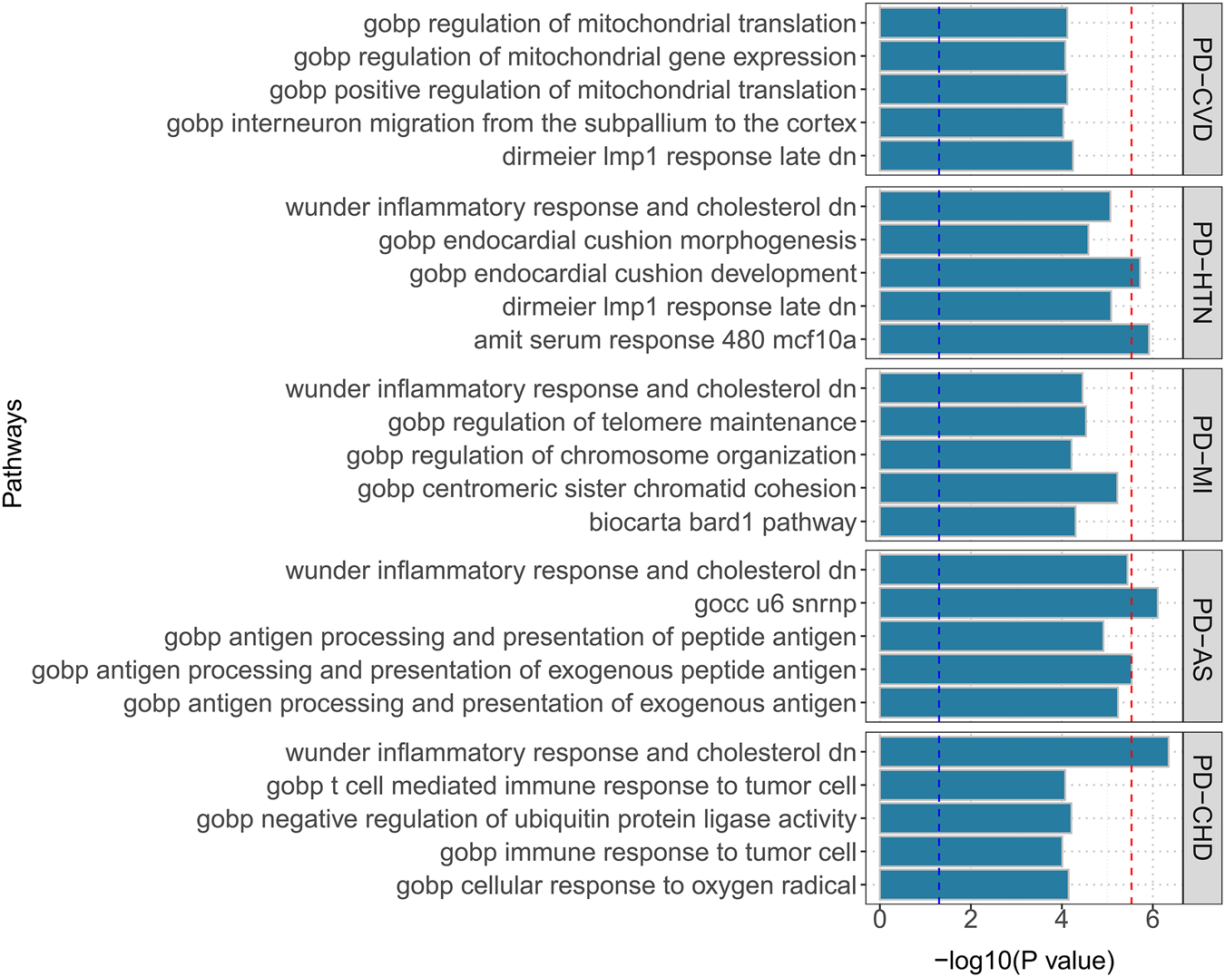
Bar plot of MAGMA gene-set analysis for genome-wide pleiotropic results. The red dotted line represents a significance of 0.05 after multiple corrections, and the blue line represents a significance of 0.05. PD Periodontitis, CVD Cardiovascular disease, HTN Hypertension, MI Myocardial Infarction, AS Atherosclerosis, CHD Coronary heart disease.

Approximately 10,678 gene sets from the MSigDB were subjected to MAGMA gene-set enrichment analysis, revealing that the inflammation-metabolism regulatory axis is a pivotal hub in the genetic crosstalk between periodontitis and five types of CVD. Notably, inflammatory response and cholesterol metabolism pathways were implicated across all four examined trait pairs, involving mechanisms of lipid oxidation, inflammasome activation, and endothelial dysfunction (Fig. 4; Supplementary Table 13). MAGMA tissue enrichment analysis revealed that risk loci for periodontitis and MI were significantly enriched in Epstein-Barr virus (EBV)-transformed lymphocytes (Supplementary Fig. 14). Intriguingly, risk loci for periodontitis with HTN (*P* = 0.057) and AS (*P* = 0.058) also showed enrichment in this tissue, approaching but not reaching statistical significance. Additionally, risk loci shared between periodontitis and AS were enriched in the coronary artery and pituitary gland (Supplementary Table 14). Adjacent genes to these risk loci (e.g.*, POLR2L, RPLP2, and DHCR24)* demonstrate differential expression in EBV-transformed lymphocytes. At the same time, *PNPLA2* exhibits significant expression differences in the spleen and whole blood tissues, respectively (Supplementary Fig. 15).

**Fig. 5:**
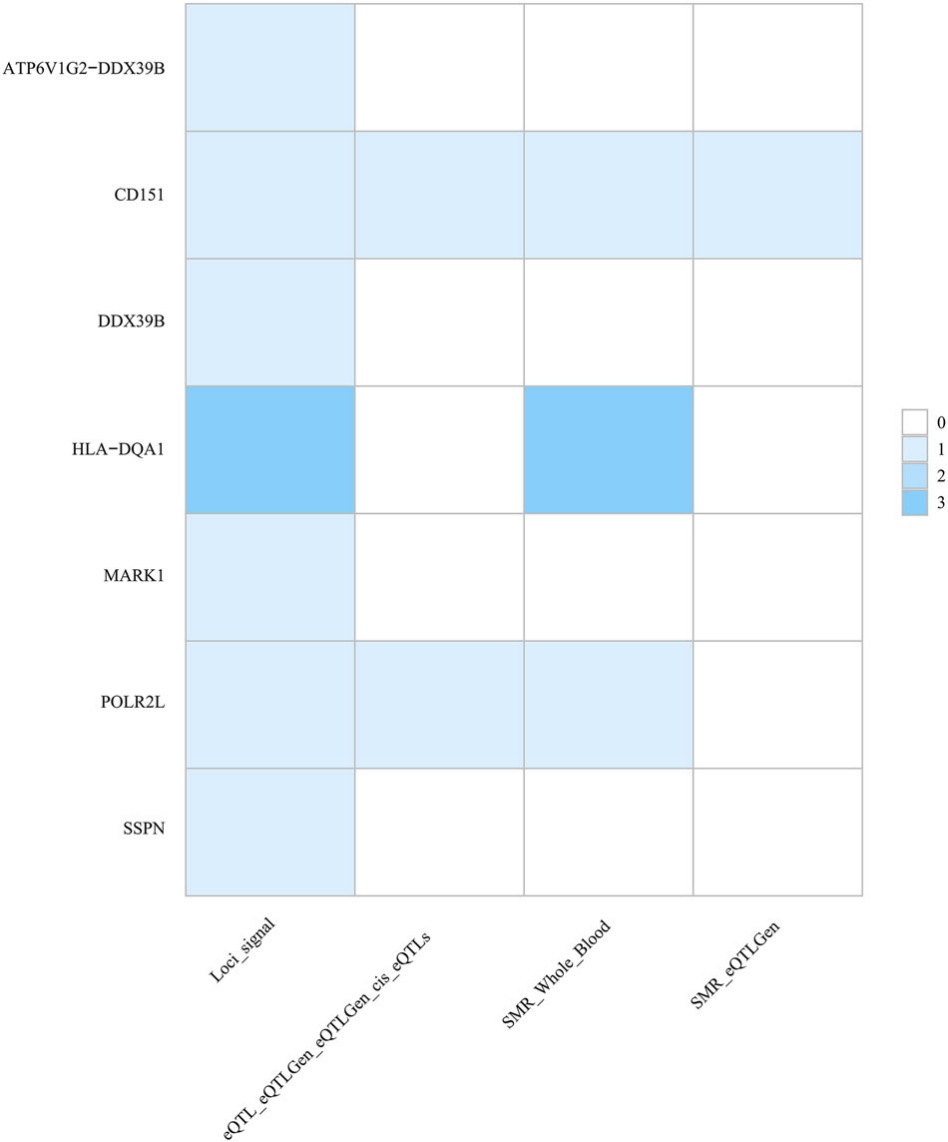
Overview of potential drug targets for periodontitis and CVD. eQTL expression quantitative trait loci, SMR summary-based Mendelian randomization.

Signals identified at the SNP level were mapped to the gene level using distinct methodological approaches. MAGMA gene analysis revealed seven statistically significant pleiotropic genes (*P* < 6 × 10^−6^, 0.05/7482), among which HLA-DQA1 was identified as a shared pleiotropic gene across three trait pairs (HTN, CHD, and AS) (Supplementary Table 15). Crucially, tissue-specific eQTL analysis revealed that the pleiotropic gene (*HLA-DQA1*) manifests pronounced expression quantitative trait effects in immunologically active tissues (EBV-transformed lymphocytes: rs200474289; Beta = −1.500; *P* = 2 × 10^−13^, spleen: rs35030446; Beta = −1.000; *P* = 3 × 10^−8^) and pathologically relevant sites (coronary artery: rs796679301; Beta = 0.510; *P* = 9 × 10^−10^, left ventricle: rs17843604; Beta = −0.570; *P* = 6 × 10^−38^, minor salivary gland: rs374459803; Beta = −0.690; *P* = 5 × 10^−09^). The remaining six genes similarly exhibited comparable tissue specificity (Supplementary Table 16). SMR analyses revealed statistically significant associations of *CD151* and *POLR2L* with cis-eQTL and whole blood. *HLA-DQA1* exhibited prominent expression in whole blood tissue (Fig. 5; Supplementary Tables 17, 18). Tissue enrichment analysis further demonstrated that these genes were significantly enriched in EBV-transformed lymphocytes, indicating that shared mechanisms between periodontitis and CVD may involve specific tissue types (Supplementary Fig. 16).

The enrichment of SNP heritability for periodontitis and five types of CVD at the tissue level was evaluated using S-LDSC analysis. Significant enrichment was identified in 13 distinct brain regions for the five types of CVDs, whereas SNPs associated with periodontitis showed enrichment in the cerebellum and frontal cortex. Additionally, SNPs linked to periodontitis and AS exhibited specific enrichment in the atrial appendage and left ventricle, respectively. (Supplementary Fig. 17 & Table 19). HyPrColoc analysis explored relationships between multiple immune cell phenotypes (e.g., B cells, T cells, myeloid cells, and CD cells) and periodontitis-CVD. Unfortunately, no immune cell phenotype exceeded the posterior probability threshold of 0.7 in HyPrColoc detection. (Supplementary Table 20).

## Discussion

Epidemiological studies have demonstrated significant associations between periodontitis and CVD in terms of incidence, progression, and complications [49–51]. However, prior literature remains inconsistent in causal conclusions [11, 52]. Given this complex interplay, this study employed a multidimensional genetic approach to systematically analyze shared genetic foundations and potential causal links, providing a theoretical basis for clinical interventions.

While previous studies have suggested a causal relationship between CVD and periodontitis, our systematic MR analysis found no evidence to support direct causation [53]. A previous randomized controlled trial involving 101 hypertensive patients with moderate/severe periodontitis demonstrated that periodontal therapy significantly reduced mean systolic blood pressure (mean difference: −11.1 mmHg; 95% CI = 6.5–15.8; *P* < 0.001), and this reduction was associated with improved periodontal status [54]. However, in this study, only the MRConMix method indicated a statistically significant association between periodontitis and HTN, albeit with wide confidence intervals. We, therefore, reasonably infer that inflammatory factors play a critical role, and periodontal therapy improves blood pressure by reducing systemic inflammatory factor levels [55]. Li et al. established a causal association between myocardial infarction and osteoporosis, postulating that inflammatory mediators may serve as substantial intermediary factors [56]. Genetic correlation analysis using LDSC and HDL revealed significant genetic correlations between periodontitis and multiple CVDs, providing robust evidence for shared genetic mechanisms.

This study identified a series of shared genetic risk loci for periodontitis and five types of CVD, among which 15q25.1 (AS) and 4p14 (CHD) demonstrated significant colocalization signals. Within the 15q25.1 region, the *CHRNA5-CHRNA3-CHRNB4* gene cluster is associated with neuronal nicotinic acetylcholine receptors regulating nicotine addiction [57]. Lutz et al. reported that 15q25.1 is significantly associated with thoracic aortic calcification, and this genetic risk is mediated by smoking [58]. Smoking, as a shared high-risk factor for both periodontitis and AS, establishes that the identification of the 15q25.1 locus provides avenues for interventions targeting their shared genetic susceptibility. This indicates that some of the pleiotropic signals observed in our study may reflect indirect, behavior-mediated pathways rather than direct biological links. Future studies should adjust for these confounders at the individual level to precisely distinguish direct from indirect genetic effects. The 1p32.3 region is a shared risk locus for periodontitis-CHD and periodontitis-MI trait pairs. The *LDLRAD1* gene encodes a protein at this locus that contains a low-density lipoprotein receptor class A domain and participates in cellular signal transduction and lipid metabolism regulation [59]. Functional impairment of this gene may lead to impaired lipid clearance, thereby promoting the formation of atherosclerotic plaques. Prior genomic studies have similarly reported associations between this gene and the prevalence of CHD and MI [60]. The *SCP2* gene encodes sterol carrier protein 2, facilitating cholesterol and other lipid transport and metabolic processes [61]. It is pivotal in maintaining intracellular cholesterol homeostasis and lipid metabolism [61]. Pathogenic variants in *SCP2* may increase susceptibility to CVD, AS, and metabolic syndrome [62]. The 1p32.3 locus modulates lipid metabolism and inflammatory responses through coordinated regulation of *LDLRAD1* and *SCP2* genes, thereby establishing a shared genetic foundation for comorbidities between periodontitis and CHD/MI. Moreover, this study queried the GWAS catalog for these genetic risk loci and identified associations with smoking and BMI, establishing risk factors for both periodontitis and CVD. This indirectly suggests that these loci may constitute shared genetic underpinnings for the comorbidity between periodontitis and CVD.

This study reveals that ‘inflammation-metabolism imbalance’ serves as a common mechanism linking periodontitis and CVD. Notably, inflammatory responses and the cholesterol metabolism pathway were identified across all four trait pairs. This pathway potentially bridges periodontitis and CVD by regulating cholesterol metabolism and inflammasome activation. Periodontitis-associated pathogens activate macrophages to release pro-inflammatory cytokines such as IL-6 and TNF-α while impairing reverse cholesterol transport [63, 64]. This dysregulation elevates low-density lipoprotein and reduces high-density lipoprotein, promoting atherosclerotic plaque formation. Atherosclerosis serves as the core pathological basis for MI and CHD, exhibiting a bidirectional relationship with HTN [65].

Tissue enrichment analysis revealed a significant enrichment of genetic loci associated with periodontitis-CVD trait pairs in EBV-transformed lymphocyte (EBV-LCLs), but we interpret this finding cautiously. EBV-LCLs are immortalized B-cell lines induced by Epstein-Barr virus and serve as an in vitro research tool rather than a standard physiological structure in humans [66]. In healthy individuals, EBV typically remains in a latent state under immune control; only upon immune dysregulation does it lead to cellular transformation and tumorigenesis [67]. Despite these limitations, the observed gene enrichment in EBV-LCLs may reflect shared mechanisms between the two diseases involving immune-mediated pathways and inflammatory responses. Further analysis using S-LDSC indicated that the SNP heritability enrichment for these traits in brain and cardiac tissues, providing more directed pathophysiological insights.

The concurrent enrichment of genetic signals in immune-relevant tissue (EBV-LCLs) and disease-targeted anatomical sites (arteries and heart) supports a bimodal mechanism hypothesis: systemic immune dysregulation may occur initially, whose local effects at the vascular level then accelerate the atherosclerotic process, ultimately contributing to the clinical co-occurrence of periodontitis and cardiovascular diseases. However, the Hyprcoloc colocalization analysis in this study failed to detect key immune cell phenotypes. This may be attributed to the complexity of biological mechanisms. Immune pathways are likely regulated by multiple genes acting synergistically rather than driven by a single strong-effect causal variant. Hyprcoloc’s assumption of a single causal variant may not adequately capture such polygenic mechanisms [68]. Furthermore, immune cell phenotypes may colocalize with disease genes only in specific developmental stages (e.g., acute inflammatory phases) or tissues (e.g., local gingival immune environments), which existing datasets have not yet encompassed.

To provide genetic support for clinical therapeutic development and reduce drug toxicity/side effects in precision medicine, we assessed the druggability potential of these risk genes. SMR analysis revealed significant colocalization of *HLA-DQA1*, *CD151*, and *POLR2L* in whole blood tissue, suggesting their mediation via immune cells, endothelial cells, or platelets; this necessitates validation through single-cell RNA sequencing to pinpoint specific cellular mechanisms. Concurrently, eQTL analyses demonstrated significant cis- and trans-regulatory effects on *CD151* and *POLR2L*. Although our results are hypothesis-generating, the exact biological functions of these genes require future functional validation. *CD151*, as a tetraspanin transmembrane protein, features well-defined extracellular domains amenable to antibody or small molecule targeting. Studies further demonstrate that *CD151* gene overexpression activates the PI3K/Akt/eNOS pathway, resulting in a 35% increase in micro-vessel density and improved cardiac function recovery [69]. The *HLA-DQA1* gene, a critical component of the human leukocyte antigen complex, plays a pivotal role in immune regulation by mediating defense responses against pathogens and influencing susceptibility to certain autoimmune disorders and pharmacogenetic reactions [70]. However, its high polymorphism poses significant challenges for direct therapeutic targeting. *POLR2L*, an essential subunit of RNA polymerase II, regulates gene transcription; direct targeting of this subunit may induce substantial adverse effects due to global transcriptional disruption [71]. Collectively, these genetic findings position *CD151* as a promising candidate for future pharmacological investigation. The well-defined extracellular domains of the *CD151* protein render it theoretically tractable for antibody-based or small-molecule targeting. However, this hypothesis requires rigorous validation, including the identification of the specific cell types (e.g., immune cells, endothelial cells, or platelets) through which it mediates risk, followed by functional studies to establish a causal role in the disease mechanisms.

This study has several limitations. First, the use of summary-level rather than individual-level GWAS data precludes stratified analysis by demographic factors and adjustment for potential confounders. Consequently, the identified pleiotropic loci and genes may not fully represent direct biological links. Second, due to the nature of summary data, individuals with comorbid periodontitis and CVD could not be excluded. Phenotypic overlap may partially drive the observed genetic associations, making it unclear whether these variants exert independent effects in individuals without comorbidity. Finally, the analysis was restricted to summary-level GWAS data from European-ancestry populations, and the sample size for immune cell-related data was limited. While this controls population stratification confounding, it limits generalizability to other groups due to ancestral differences in allele frequencies, LD patterns, and gene-environment interactions. Future studies utilizing individual-level data, incorporating detailed phenotypic covariates or mediation models, and validating findings in disease-specific cohorts will help clarify the underlying mechanisms.

### Ethical approval and consent

The original study obtained all necessary ethical approvals and participant consent. Therefore, no additional approval was needed for this study of data.

## Conclusion

Building on the close epidemiological link between periodontitis and CVD, this study systematically investigated their shared genetic architecture. The results revealed that the relationship was not causal but stems from shared genetic predisposition and pathophysiology. This study identified shared pleiotropic risk loci (e.g., 15q25.1, 4p14, and 1p32.3) and genes (e.g., *HLA-DQA1*, *CD151*, and *POLR2L*), further implicating key pathways involved in inflammatory response and cholesterol metabolism. However, the interpretation of these findings warrants caution. Further research utilizing individual-level data to adjust for confounders and functional validation studies were needed to clarify the precise mechanisms underlying these genetic links.

## Supplementary information

Supplementary Table

Supplementary Figure

## Data Availability

Data are available in public, open-access repositories corresponding to the original studies (FinnGne https://www.finngen.fi/en/access_results; GLIDE https://data.bris.ac.uk/data/dataset). The data that support the findings of this study are available from the corresponding author upon reasonable request.
