# Supplementary material for: Exploring the shared genetic architecture between periodontitis and cardiovascular disease
Source: BDJ Open. 2026 Mar 31;12:28. doi: 10.1038/s41405-026-00421-4 (PMC13039211; doi:10.1038/s41405-026-00421-4)
Supplement: Supplementary file 2 — Supplementary Figure [file 41405_2026_421_MOESM2_ESM.docx]

**Supplementary materials**

**Supplementary Methods**

**Supplementary Figure**

**Figure 1.** Flow chart describing the screening of valid instrumental variable selection

**Figure 2.** Sensitivity analysis of MR estimates on the association of periodontitis with CVD

**Figure 3.** Sensitivity analysis of MR estimates on the association of CVD with periodontitis

**Figure 4.** MVMR studies investigate the causal relationships between CVD and periodontitis after adjusting for Smoking, BMI, and T2D

**Figure 5.** Manhattan plot of the PLACO results

**Figure 6.** QQ plots for pleiotropic results performed by PLACO

**Figure 7.** Sensitivity Analysis for Bayesian Colocalization.

**Figure 8.** Regional plots of each colocalized locus were identified for the corresponding trait pair (PD-CVD) by using the PLACO

**Figure 9.** Regional plots of each colocalized locus were identified for the corresponding trait pair (PD-HTN) by using the PLACO

**Figure 10.** Regional plots of each colocalized locus were identified for the corresponding trait pair (PD-MI) by using the PLACO

**Figure 11.** Regional plots of each colocalized locus were identified for the corresponding trait pair (PD-AS) by using the PLACO

**Figure 12.** Regional plots of each colocalized locus were identified for the corresponding trait pair (PD-CHD) by using the PLACO

**Figure 13.** Gene set enrichment analysis of phenotypes associated with pleiotropic genes shared between PD and CVD

**Figure 14.** Bar plot of MAGMA tissue analysis for genome-wide pleiotropic results

**Figure 15.** Heatmap for expression values of pleiotropic genes in different tissues

**Figure 16.** Gene enrichment for identified pleiotropic genes

**Figure 17.** Heatmap of tissues shared between periodontitis and cardiovascular diseases identified by S-LDSC.

**Supplemental Methods**

**Detailed information of Mendelian Randomization (MR) methods**

***F statistic***. In order to measure the strength of instruments, *F* statistic is constructed by formula:

$$F=(\frac{n-1-k}{k})(\frac{R^{2}}{1-R^{2}})$$

where R^2^ represents the proportion of variance explained by genetic variants, *n* represents sample size, *k* represents number of SNPs.

##### Pleiotropic analysis under composite null hypothesis analysis (PLACO)

Pleiotropic signal detection was performed by employing summary-level association statistics across complex traits. This involved calculating a Z-score correlation matrix, followed by the application of a level-α intersection-union test (IUT) to evaluate the pleiotropy hypothesis: *H*_0_ is the null hypothesis, which could be expressed as $H_{0}:H_{00}\cup H_{01}\cup H_{02}$, and alternative hypothesis *H*_1_ could be further expressed as:

$$\begin{matrix} H_{1}:H_{00}^{a}\cap H_{01}^{a}\cap H_{02}^{a}\text{, } \\ \text{where }H_{00}:\beta_{CVD}=\beta_{Periodontitis}=0\text{,} \\ H_{01}:\beta_{CVD}=0,\text{ }\beta_{Periodontitis}\neq0\text{,} \\ H_{02}:\beta_{CVD}\neq0,\text{ }\beta_{Periodontitis}=0. \end{matrix}$$

The$H^{a}$represents the complement of *H*. $\beta_{Disease}$ represents effect size of autoimmune diseases. The maximum of *P* values for testing *H*_0_ vs *H*_1_ were viewed as the final *P* values.

##### MAGMA analysis

In MAGMA gene analysis, genetic markers are consolidated to the gene level, translating single-SNP associations into a combined effect of all gene markers on the phenotype. The analysis is built on a multiple linear principal component regression model. To manage highly correlated SNPs and ensure model identifiability, the gene's SNP matrix is reduced to its principal components (PCs). After removing PCs with negligible eigenvalues, the remaining ones are used as predictors in a linear model, and gene p-values are derived from an F-test. The same logic of aggregation extends to gene-set analysis, where genes are grouped into functionally related sets. This process curtails the multiple-testing burden and improves the ability to detect subtle, polygenic effects that individually might be weak.

##### Multi-trait colocalization analysis using HyPrColoc

HyPrColoc is built upon a statistical model similar to coloc but capitalizes on summary statistics from a large set of traits to uncover colocalization signals among them. This technique efficiently approximates the posterior probability of colocalization by considering only a small subset of putative causal associations, operating under the assumption of a single causal variant per trait. This strategy avoids the inefficiency of repeated pairwise analyses, facilitating a swift and effective search for multi-trait colocalization. A notable drawback of this approach is its potential for an elevated false-negative rate and compromised performance in identifying shared causal variants.

##### Detailed information of immune cells used in HyPrColoc analysis

Summary statistics for immune traits were sourced from the publicly accessible GWAS Catalog. This dataset originated from a GWAS of 3,757 individuals of European ancestry (57% female), which interrogated approximately 22 million single nucleotide polymorphisms (SNPs) genotyped on high-density arrays. The analysis adjusted for covariates including sex and age, and the genotypes were subsequently imputed using a sequence-based reference panel.

**Supplemental Figures**

**
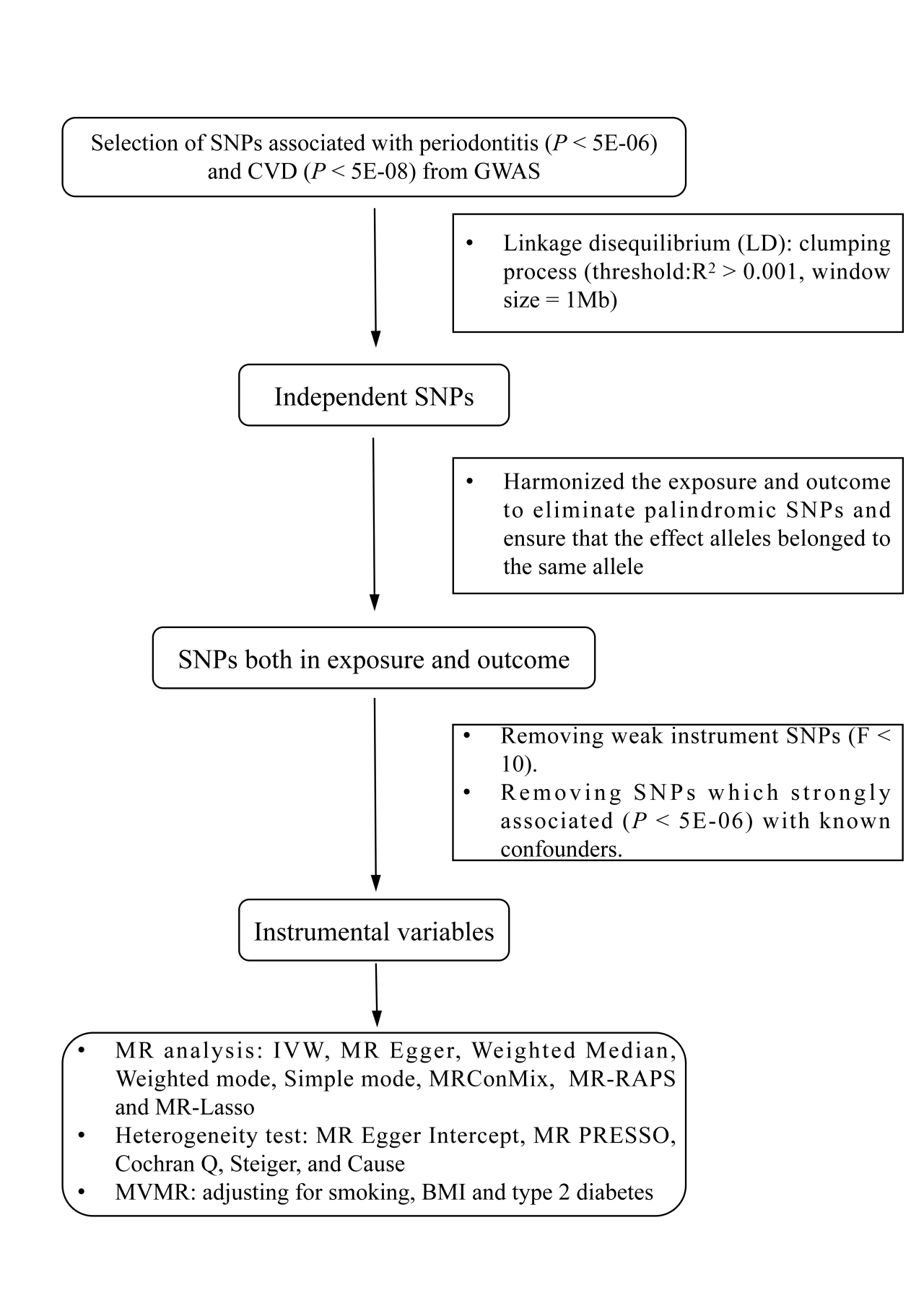
**

**Figure 1.** Flow chart describing the screening of valid instrumental variable selection.


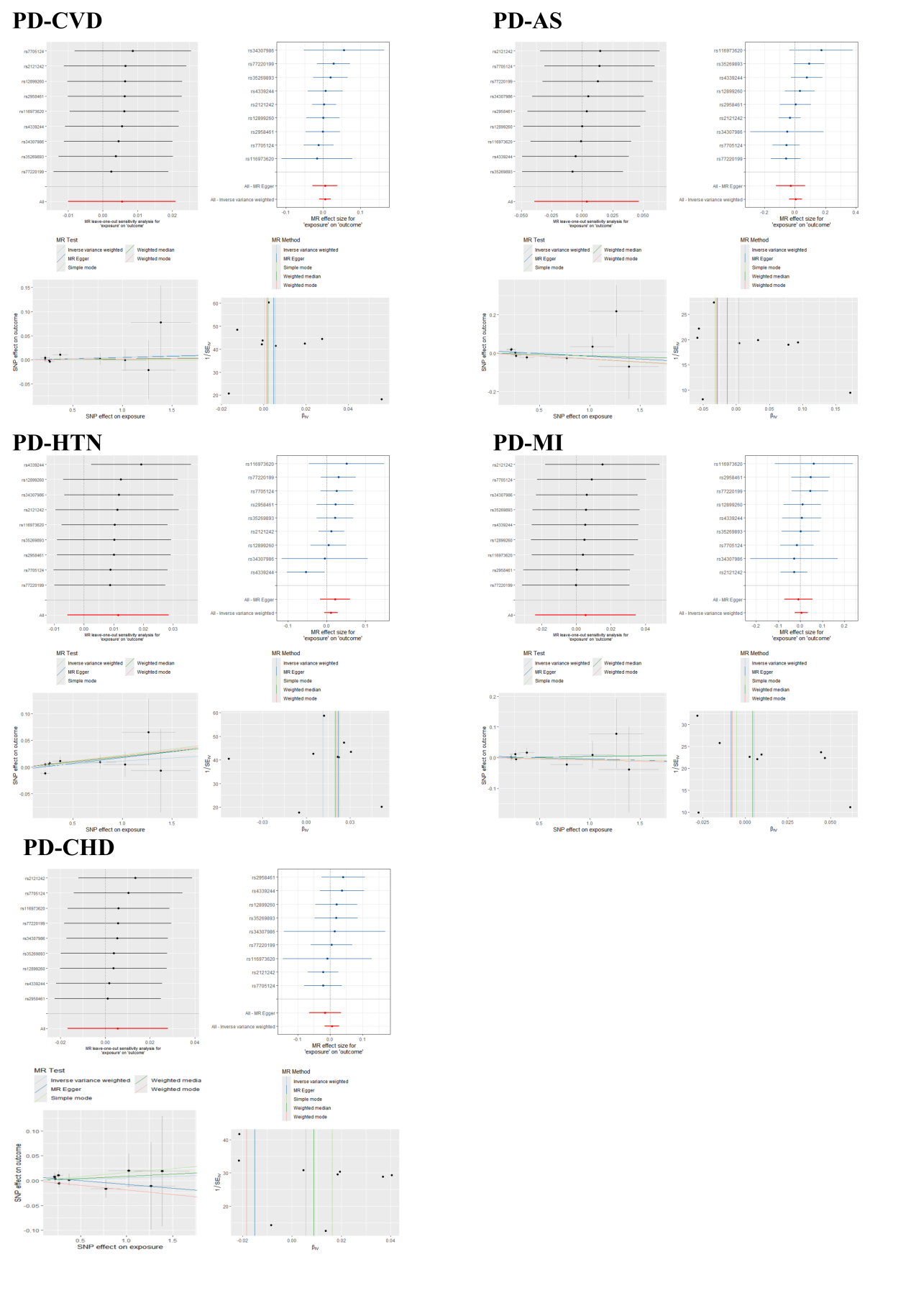


**Figure 2**. Sensitivity analysis of MR estimates on the association of periodontitis with CVD. PD, Periodontitis; CVD, Cardiovascular disease; HTN, Hypertension; MI, Myocardial Infarction; AS, Atherosclerosis; CHD, Coronary heart disease.


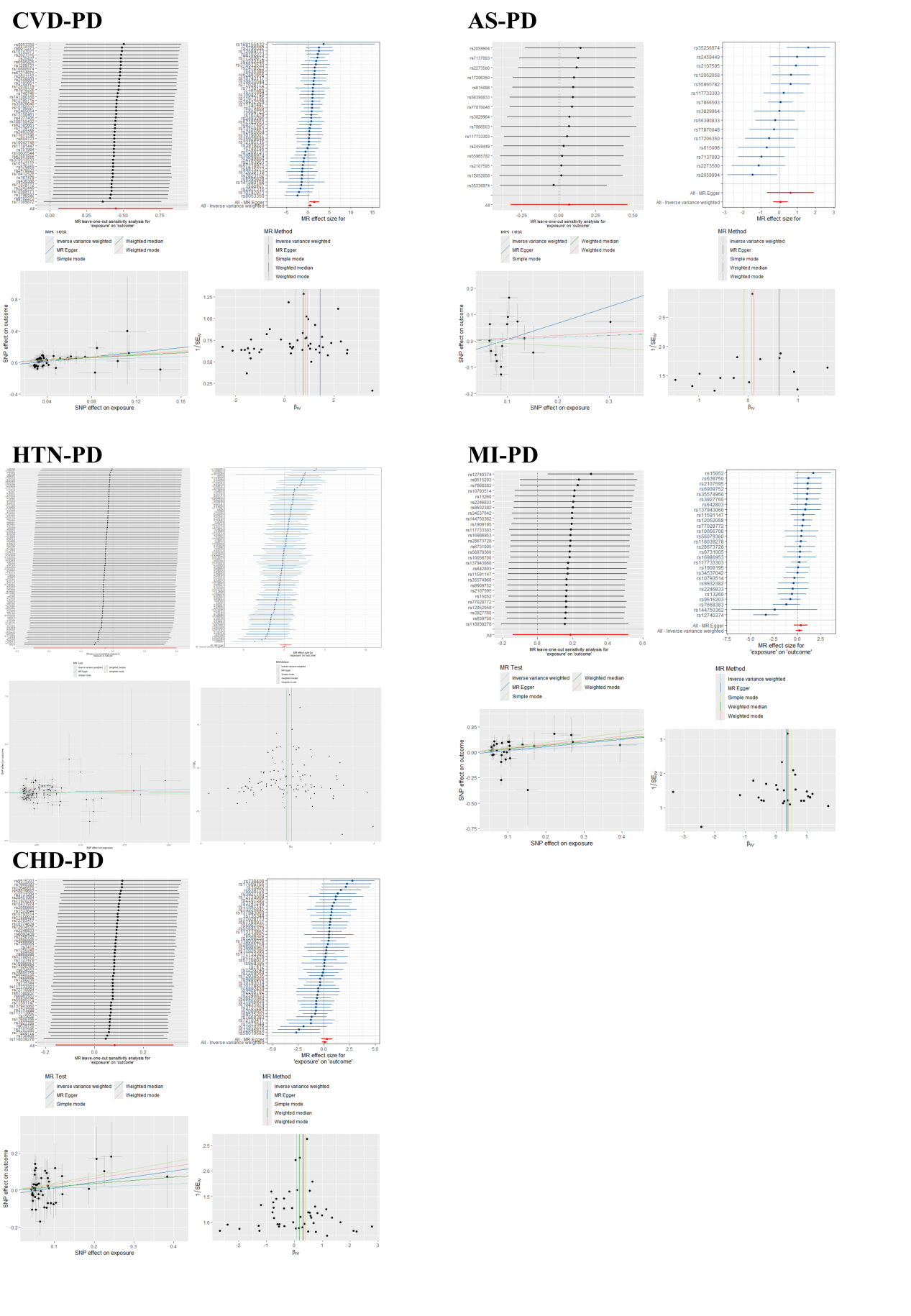


**Figure 3**. Sensitivity analysis of MR estimates on the association of CVD with periodontitis. PD, Periodontitis; CVD, Cardiovascular disease; HTN, Hypertension; MI, Myocardial Infarction; AS, Atherosclerosis; CHD, Coronary heart disease.


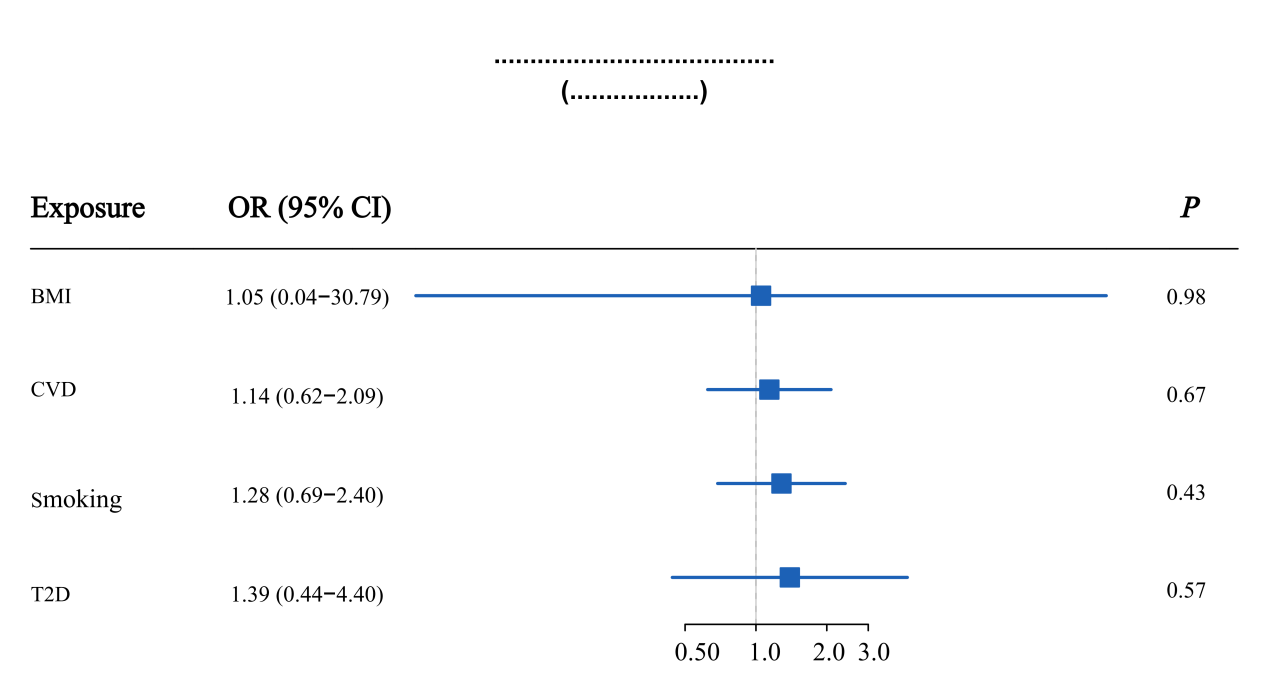


**Figure 4.** MVMR studies investigate the causal relationships between CVD and periodontitis after adjusting for Smoking, BMI, and T2D. OR, odds ratio; 95% CI, the 95% confidence interval; BMI, body mass index; T2D, type 2 diabetes.


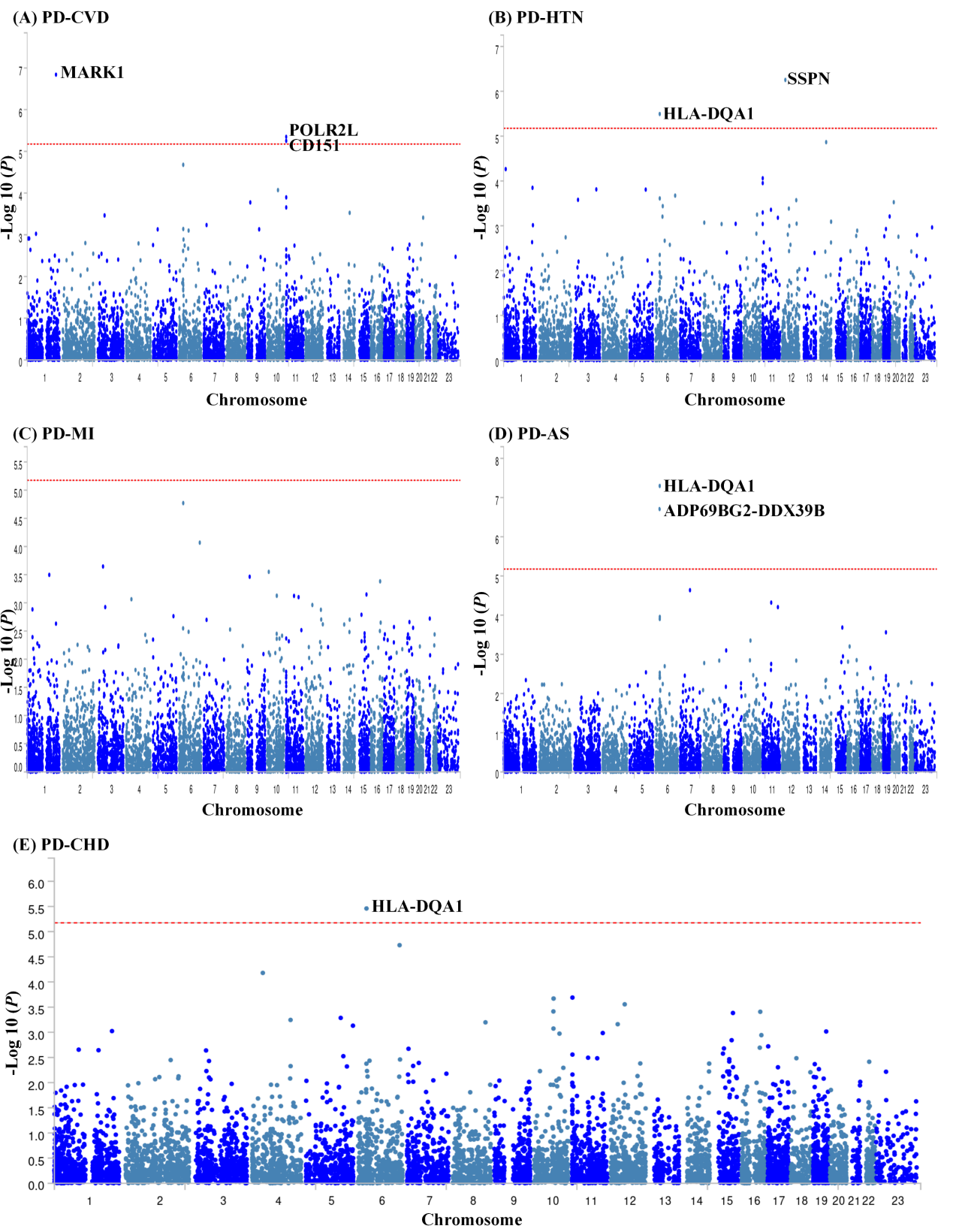


**Figure 5.** Manhattan plot of the PLACO results. Note: The red line represents the significance of 5×10^-8^. PD, Periodontitis; CVD, Cardiovascular disease; HTN, Hypertension; MI, Myocardial Infarction; AS, Atherosclerosis; CHD, Coronary heart disease.


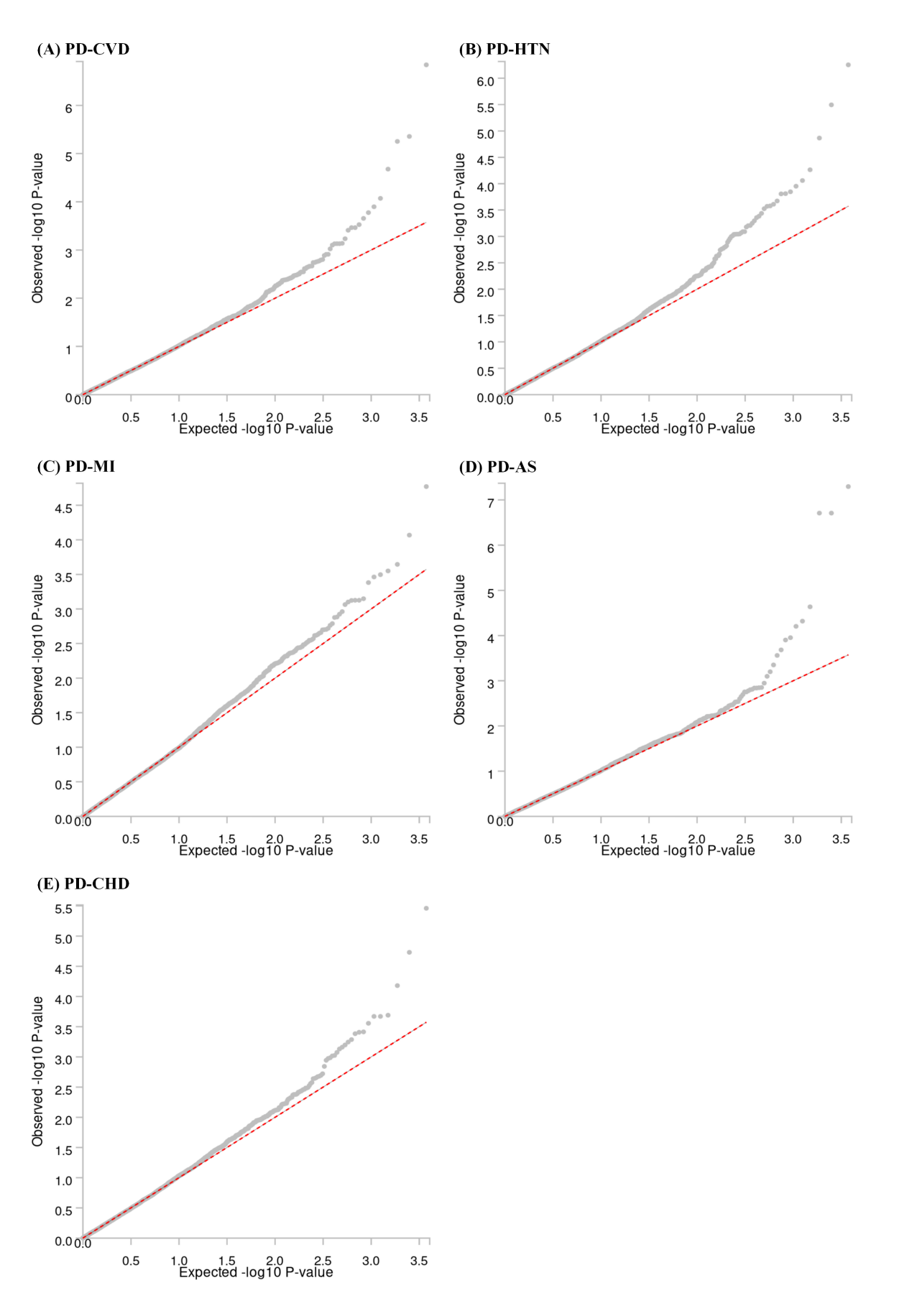


**Figure 6.** QQ plots for pleiotropic results performed by PLACO. PD, Periodontitis; CVD, Cardiovascular disease; HTN, Hypertension; MI, Myocardial Infarction; AS, Atherosclerosis; CHD, Coronary heart disease.


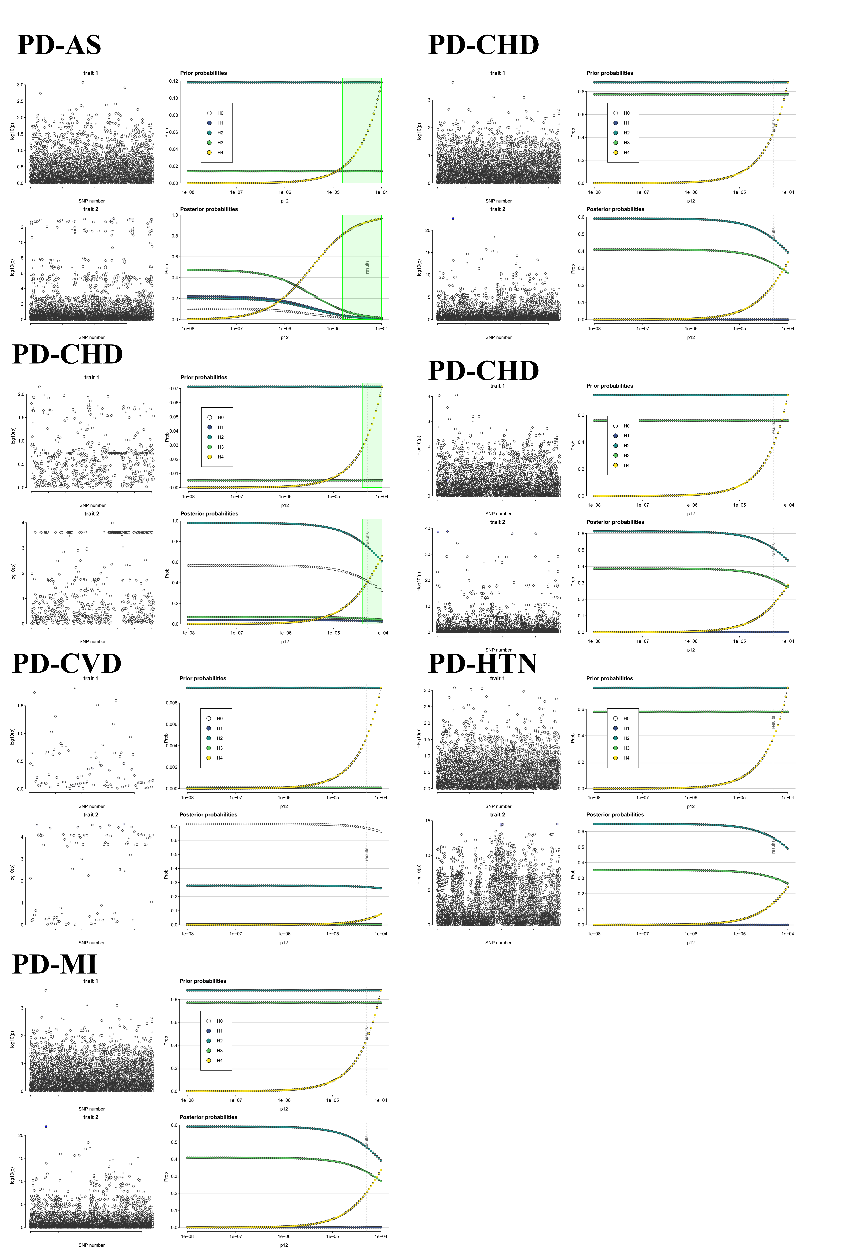


**Figure 7.** Sensitivity Analysis for Bayesian Colocalization. Note: PD, Periodontitis; CVD, Cardiovascular disease; HTN, Hypertension; MI, Myocardial Infarction; AS, Atherosclerosis; CHD, Coronary heart disease


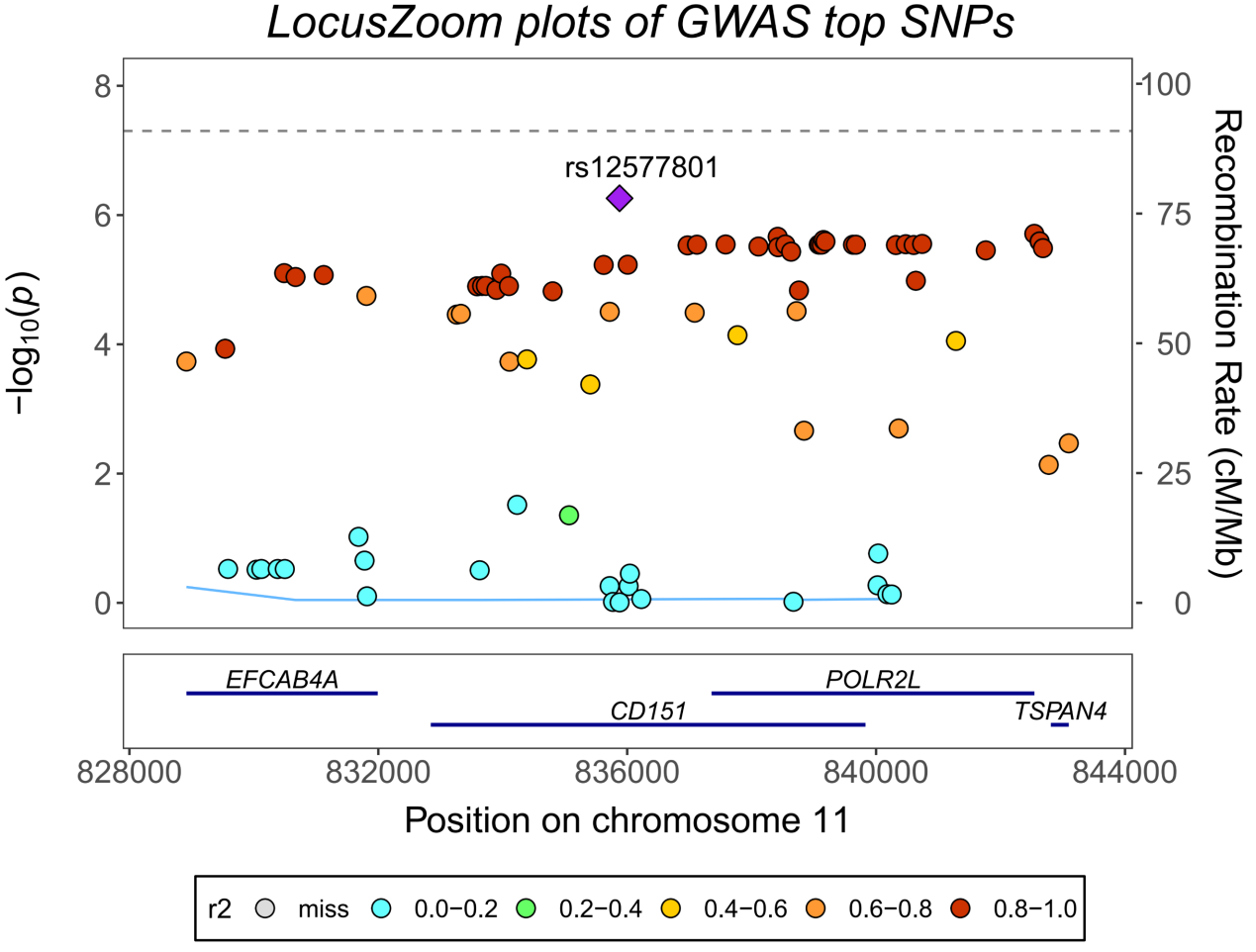


**Figure 8.** Regional plots of each colocalized locus were identified for the corresponding trait pair (PD-CVD) by using the PLACO. Note: SNPs in LD that do not have any significant independent lead SNPs in the selected region are grayed out. PD Periodontitis; CVD Cardiovascular disease; SNP, Single-nucleotide polymorphism; LD, Linkage disequilibrium.


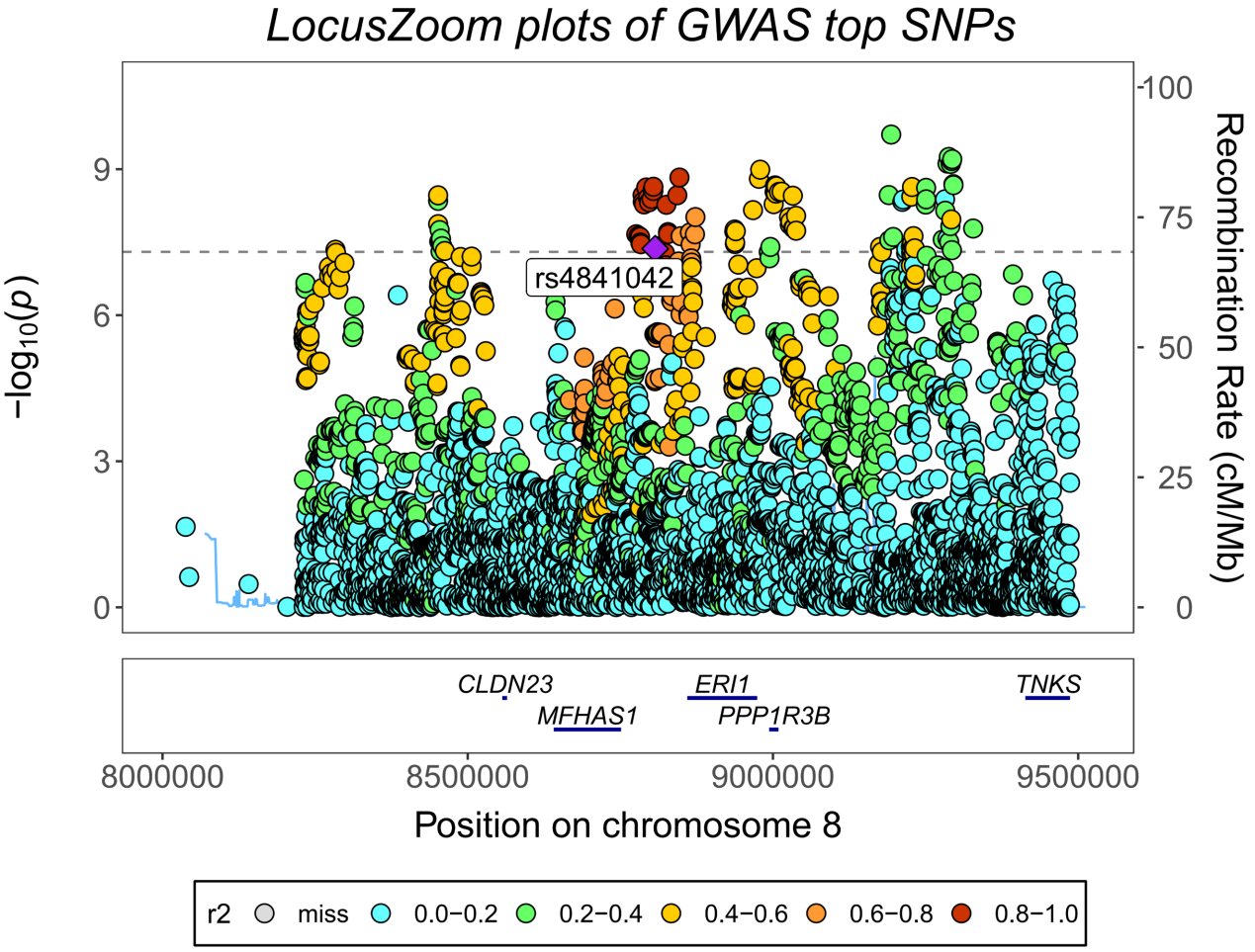


**Figure 9.** Regional plots of each colocalized locus were identified for the corresponding trait pair (PD-HTN) by using the PLACO. Note: SNPs in LD that do not have any significant independent lead SNPs in the selected region are grayed out. PD Periodontitis; HTN Hypertension; SNP, Single-nucleotide polymorphism; LD, Linkage disequilibrium.


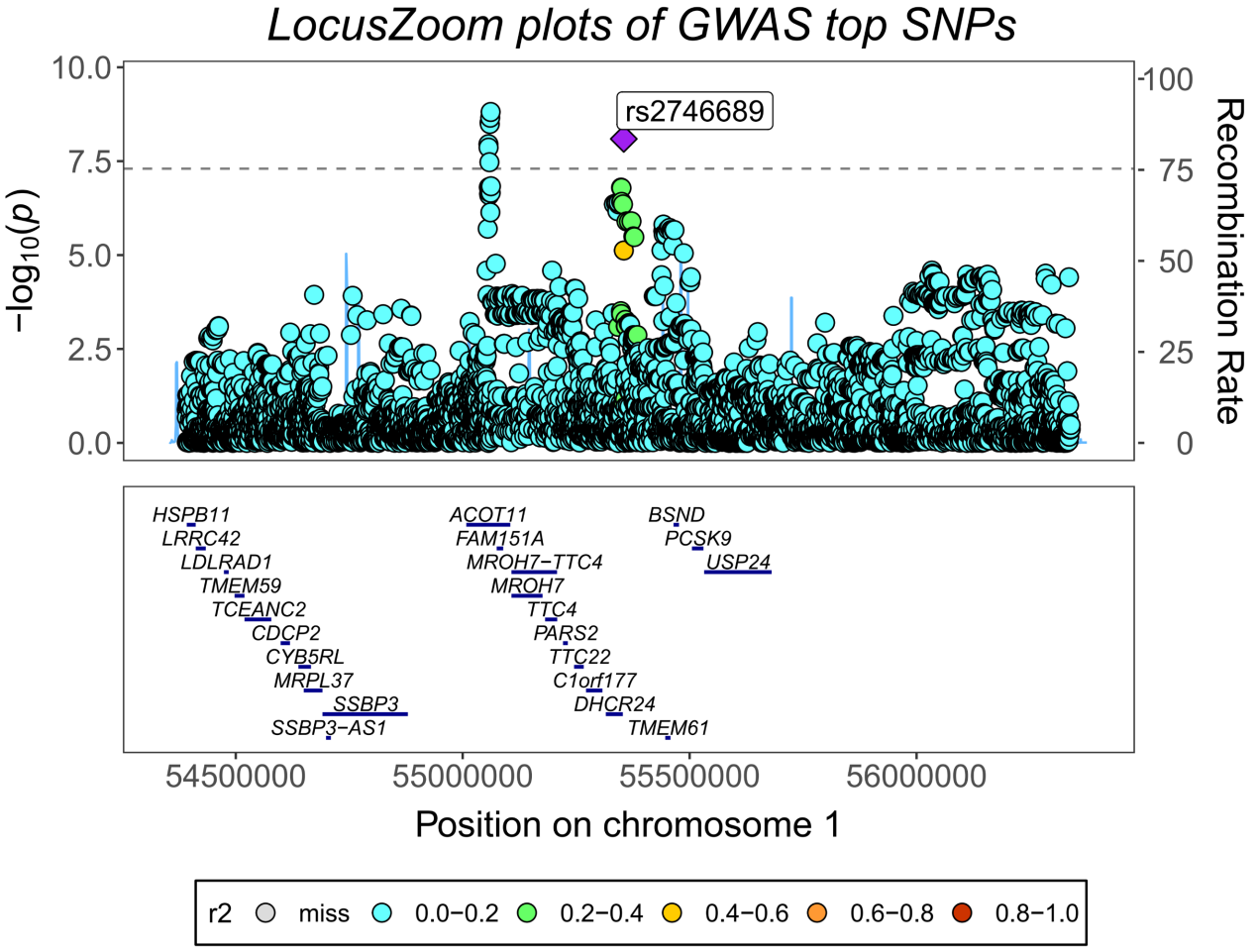


**Figure 10.** Regional plots of each colocalized locus were identified for the corresponding trait pair (PD-MI) by using the PLACO. Note: SNPs in LD that do not have any significant independent lead SNPs in the selected region are grayed out. PD Periodontitis; MI Myocardial Infarction; SNP, Single-nucleotide polymorphism; LD, Linkage disequilibrium.


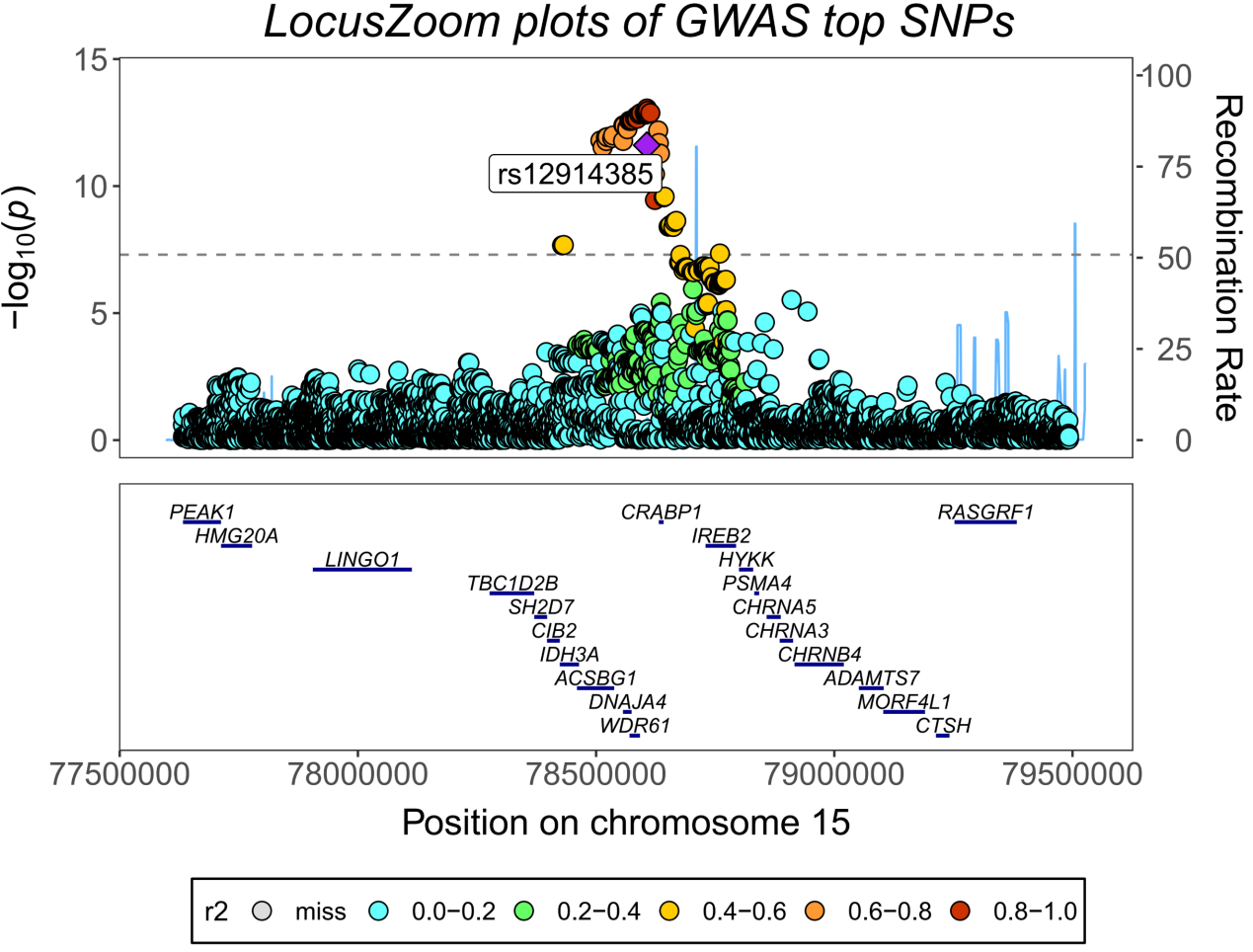


**Figure 11.** Regional plots of each colocalized locus were identified for the corresponding trait pair (PD-AS) by using the PLACO. Note: SNPs in LD that do not have any significant independent lead SNPs in the selected region are grayed out. PD Periodontitis; AS Atherosclerosis; SNP, Single-nucleotide polymorphism; LD, Linkage disequilibrium.


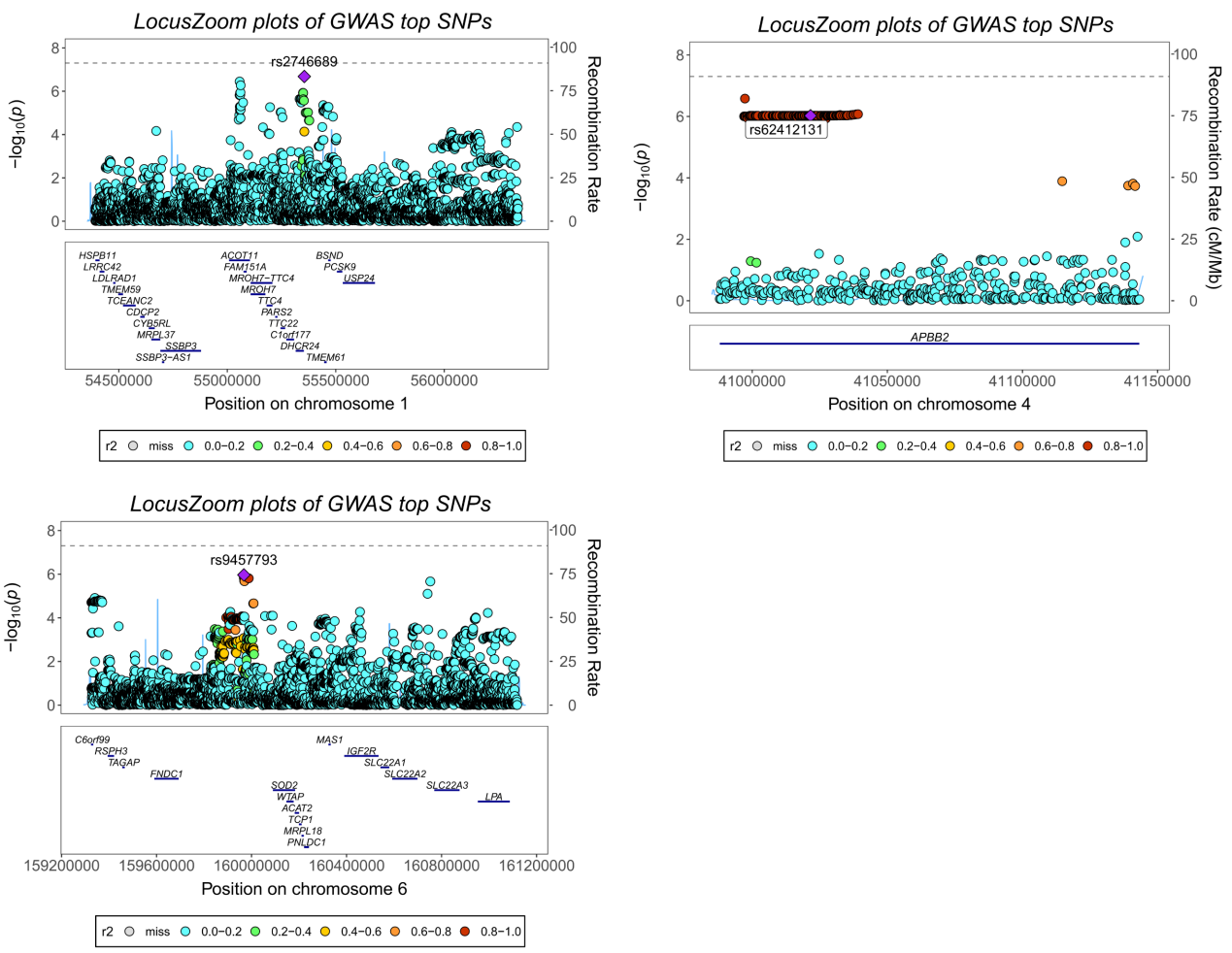


**Figure 12.** Regional plots of each colocalized locus were identified for the corresponding trait pair (PD-CHD) by using the PLACO. Note: SNPs in LD that do not have any significant independent lead SNPs in the selected region are grayed out. PD, Periodontitis; CHD, Coronary heart disease; SNP, Single-nucleotide polymorphism; LD, Linkage disequilibrium.


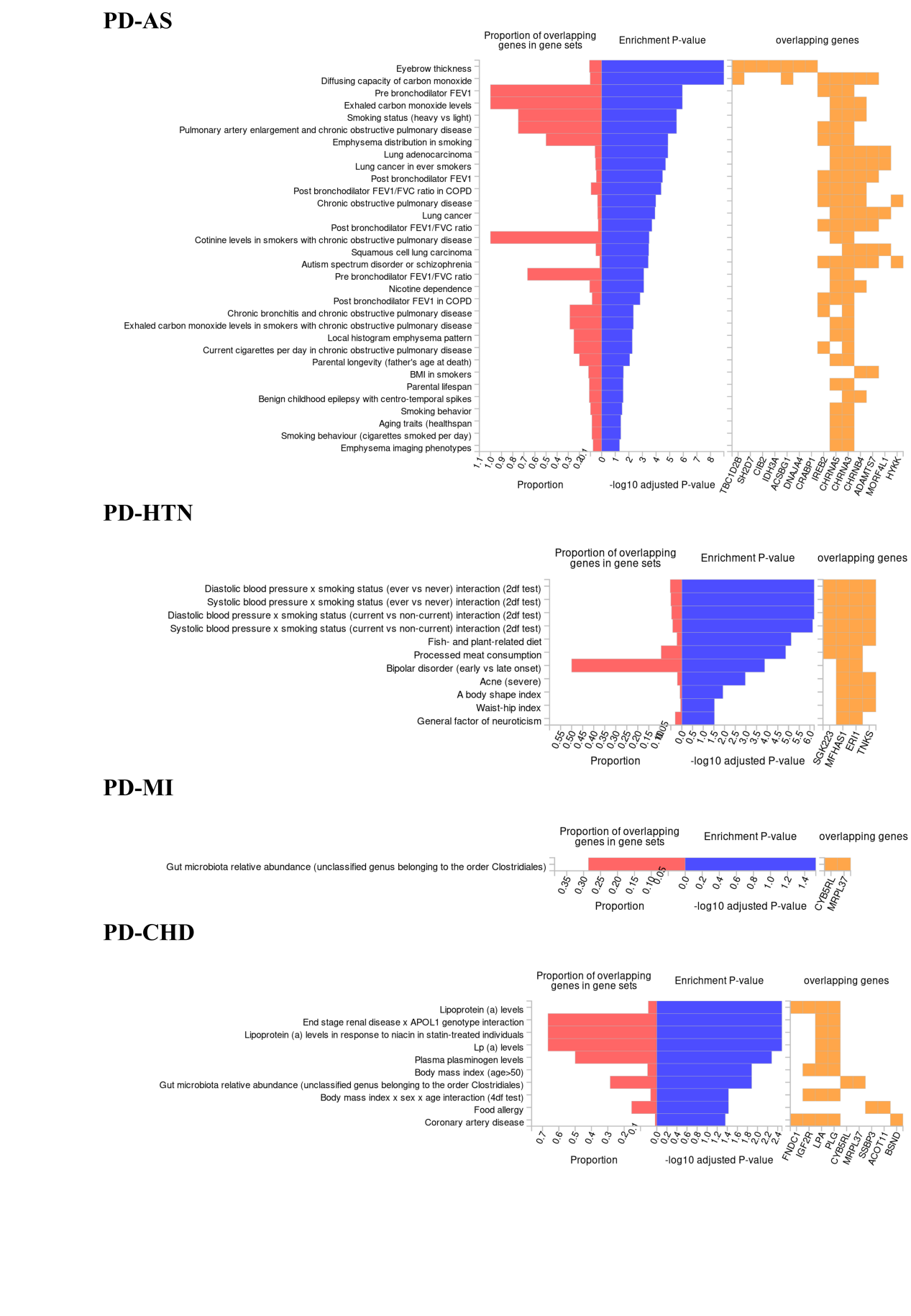


**Figure 13.** Gene set enrichment analysis of phenotypes associated with pleiotropic genes shared between PD and CVD. The left column lists the phenotypes, with the proportion of overlapping genes in blue and the enrichment -log10(p-value) in red. The right column highlights the specific overlapping genes for each phenotype. PD, Periodontitis; CVD, Cardiovascular disease; HTN, Hypertension; MI, Myocardial Infarction; AS, Atherosclerosis; CHD, Coronary heart disease.


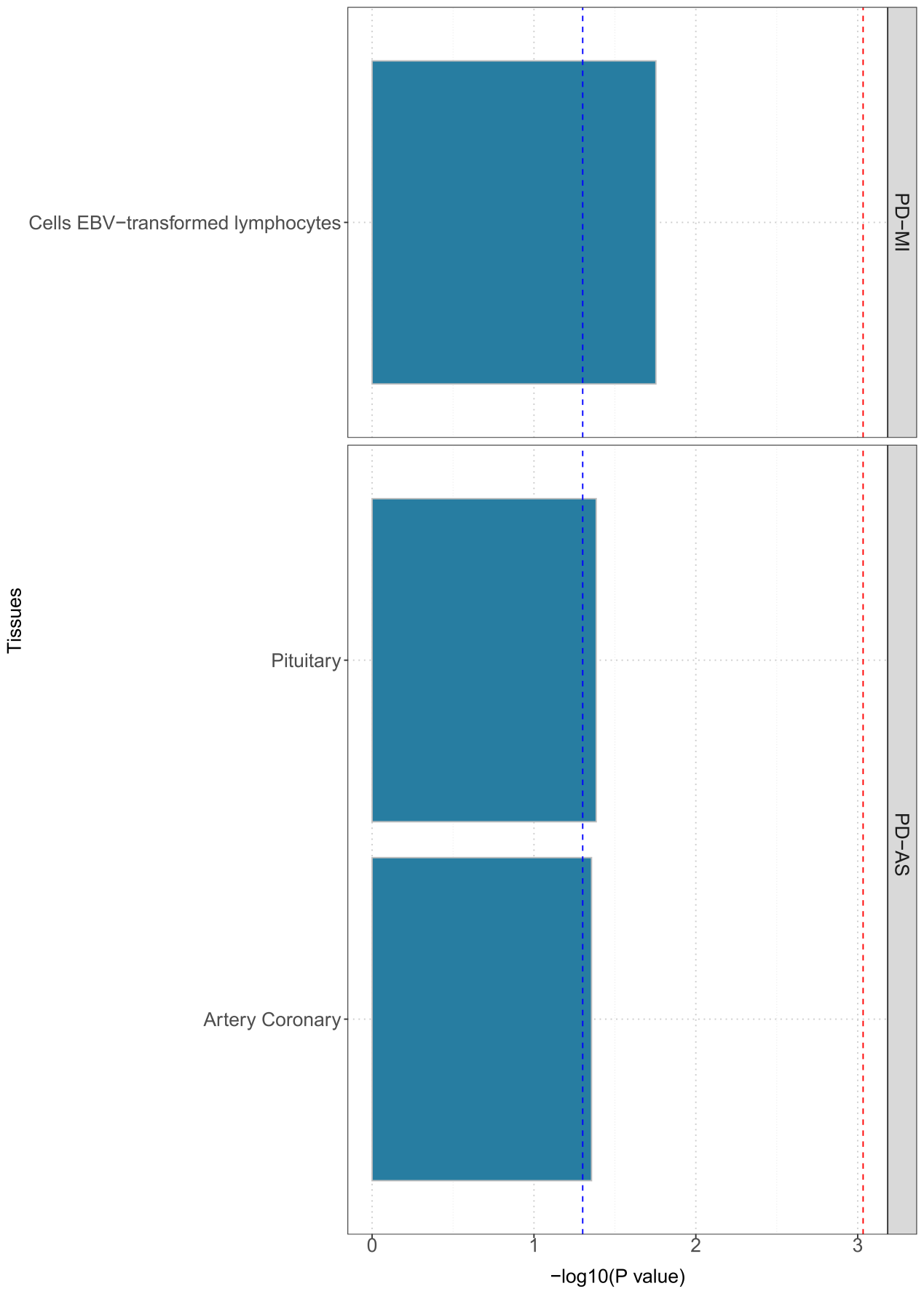


**Figure 14.** Bar plot of MAGMA tissue analysis for genome-wide pleiotropic results. Note: The red dotted line represents the significance of 0.05 after multiple corrections, and the blue represents the significance of 0.05. PD, Periodontitis; MI, Myocardial Infarction.


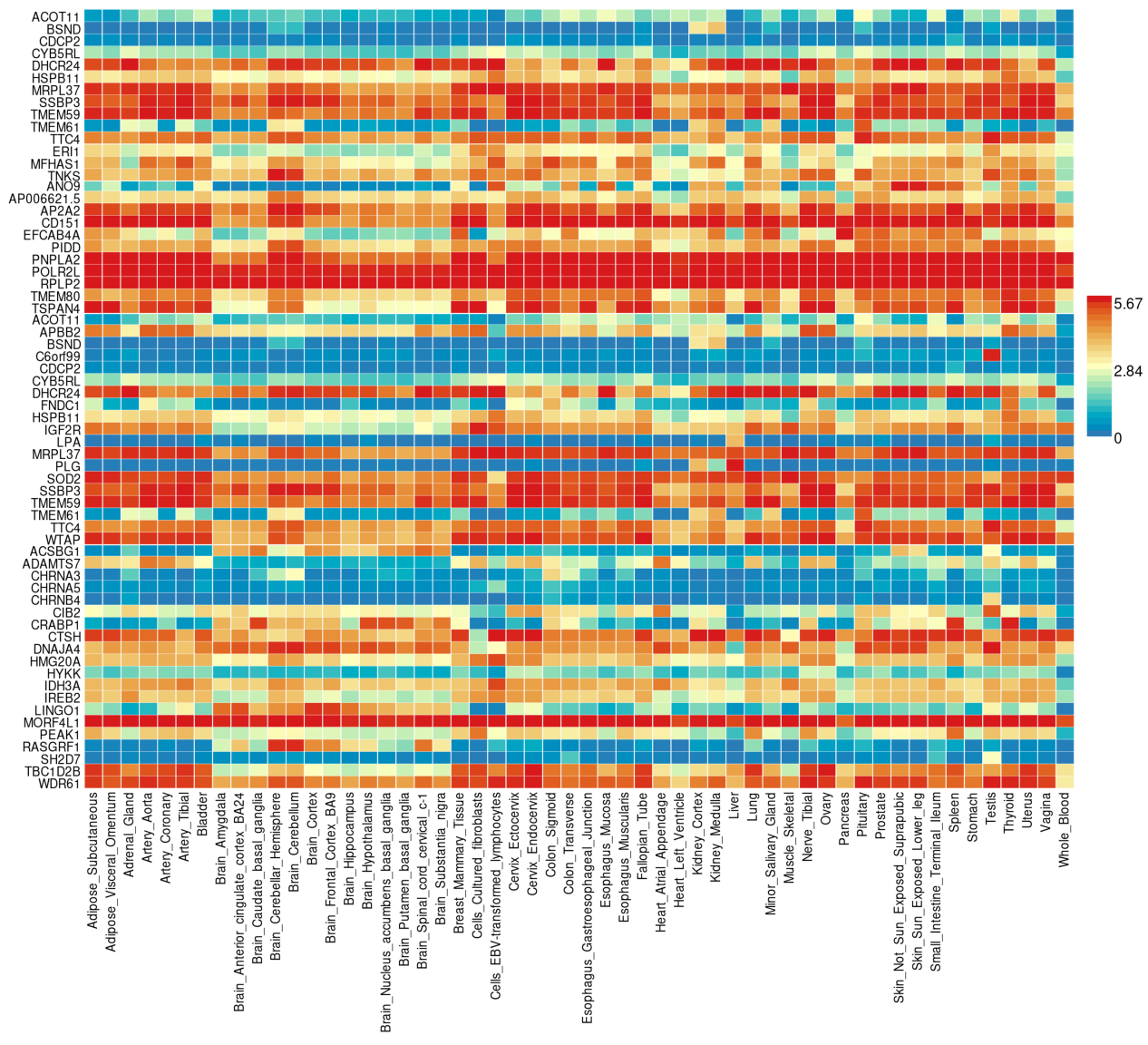


**Figure 15.** Heatmap for expression values of pleiotropic genes in different tissues.


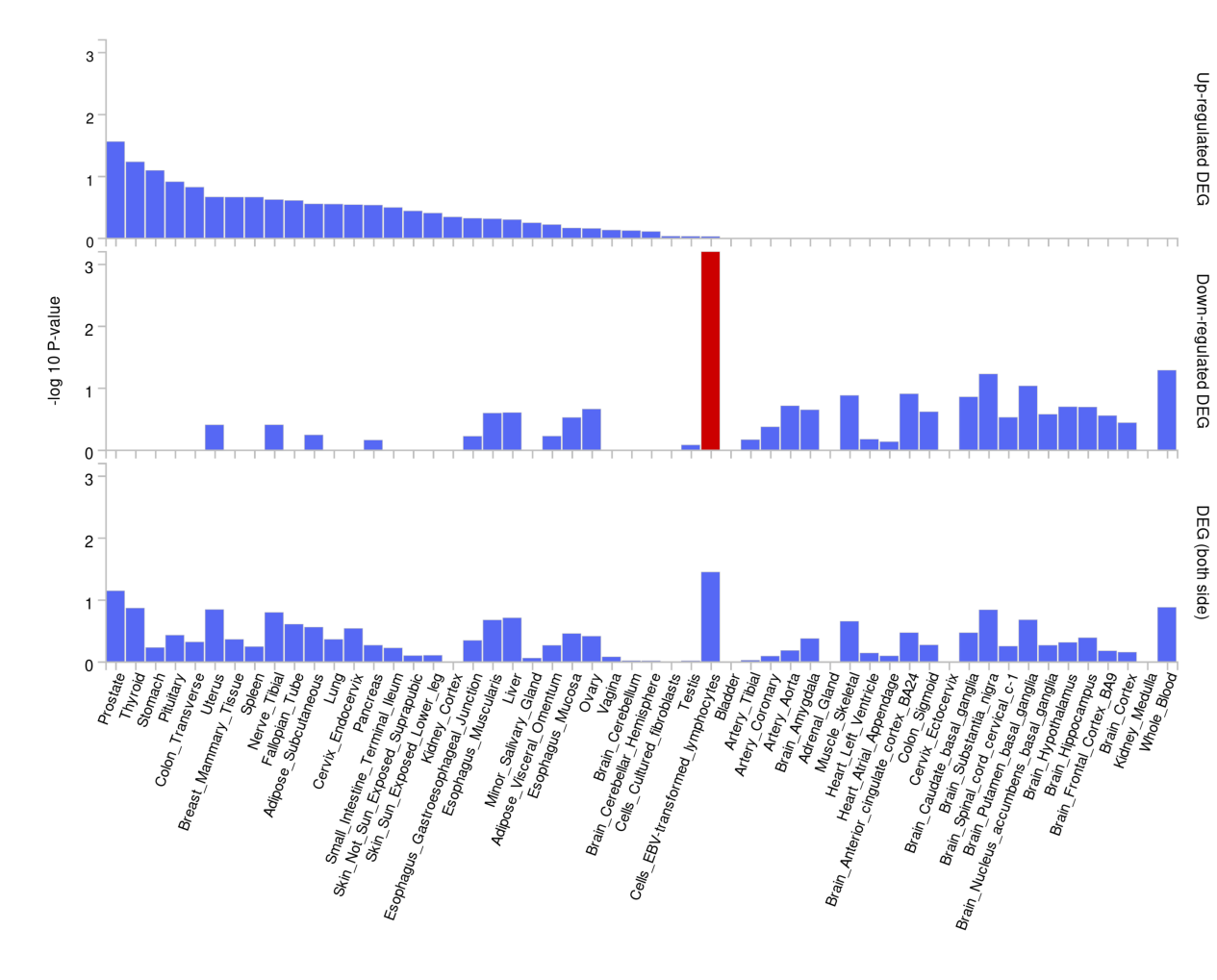


**Figure 16.** Gene enrichment for identified pleiotropic genes. Red panels represent significant tissues after Bonferroni adjustment. Abbreviations: DEG, differentially expressed genes.


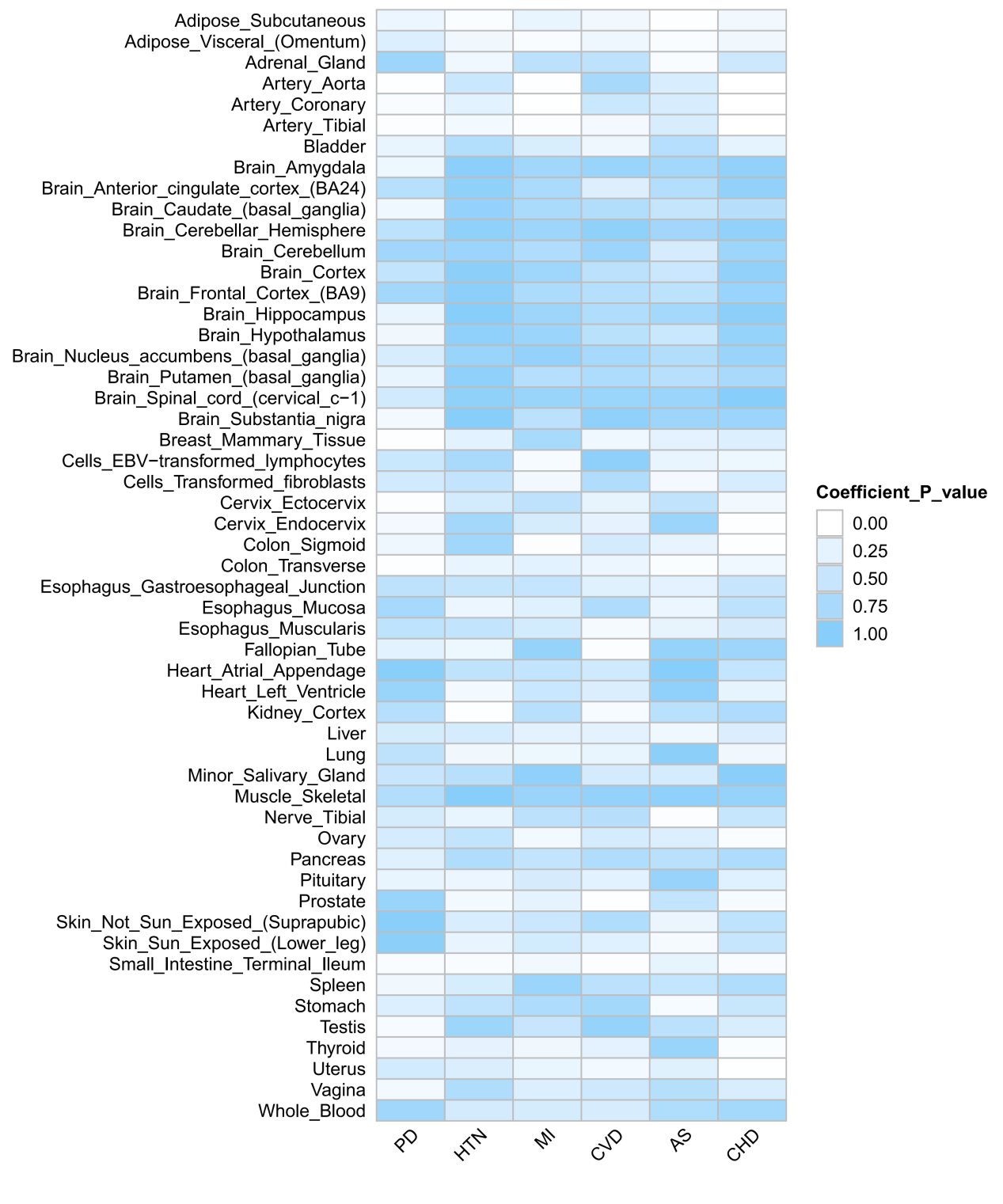


**Figure 17.** Heatmap of tissues shared between periodontitis and cardiovascular diseases identified by S-LDSC. Note: PD, Periodontitis; CVD, Cardiovascular disease; HTN, Hypertension; MI, Myocardial Infarction; AS, Atherosclerosis; CHD, Major coronary heart disease event.
